# Very Anisotropic 2D Molecular Magnetic Materials Based on Pentagonal Bipyramidal Heptacyanidorhenate(IV)

**DOI:** 10.3390/ma15238324

**Published:** 2022-11-23

**Authors:** Eufemio Moreno Pineda, Wolfgang Wernsdorfer, Kira E. Vostrikova

**Affiliations:** 1 Departamento de Química-Física, Facultad de Ciencias Naturales, Exactas y Tecnología, Universidad de Panamá, Transístmica 0874, Panama; 2 Physikalisches Institut, Karlsruhe Institute of Technology, 1 Wolfgang-Gaede-Str., D-76131 Karlsruhe, Germany; 3 Nikolayev Institute of Inorganic Chemistry SB RAS, 3 Lavrentiev Avenue, 630090 Novosibirsk, Russia

**Keywords:** 2D coordination polymers, cyanide-bridged heterometallic assemblies, heptacyanorhenate(IV), manganese(III) Schiff base complexes, orbitally degenerate *5d* complex, pentagonal bipyramidal cyanidometallate

## Abstract

The first neutral 2D heterometallic assemblies based on orbitally degenerate heptacyanidorhenate(IV) were prepared and structurally characterized. An analysis of the magnetic data for the polycrystalline samples of Ph_4_P[{Mn(acacen)}_2_Re(CN)_7_]·Solv (**1**) and PPN[{Mn(acacen)}_2_Re(CN)_7_]·Solv (**2**) have shown that both materials display slow magnetic relaxation at temperatures below 10 and 21 K for **1** and **2**, respectively. Despite the presence of the same molecular magnetic modules that make up the anionic layers, the studied 2D networks differ significantly in magnetic anisotropy, having a small coercive field (0.115 T) for **1** and a large one (~2.5 T) for **2** at 2 K. In addition, for both polymers a M(H) value does not saturate at the maximum available field of 7 T, and the material **2** is a metamagnet. This intriguing difference originates from the cooperative anisotropic spin interaction in Re^IV^−CN−Mn^III^ pairs and the zero field splitting (ZFS) effect of Mn^III^ ions with a noncollinear alignment of the local magnetic axes in crystals of the compounds.

## 1. Introduction

**Scheme 1:**
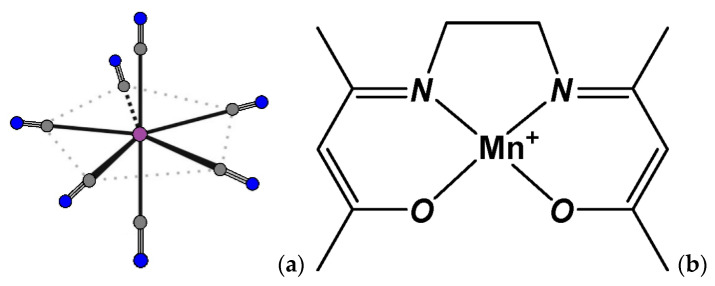
Building units: (**a**) PBP [M(CN)_7_]^3/4−^ (*D*_5h_); (**b**) [Mn(acacen)]^+^.

Although the era of molecular magnetism is almost half a century old, approaches to the targeted chemical design of such materials are still in their infancy. Orbitally degenerate cyanidometallates possessing an unquenched orbital angular momentum are efficient sources of strong magnetic anisotropy for designing the molecular magnetic materials. This was initially predicted theoretically [1,2] and then established experimentally by synthesis of a number of single-molecule magnets (SMMs) [3,4,5,6,7,8,9,10] and single-chain magnets (SCMs) [11,12,13,14,15,16,17,18,19,20] involving the homoleptic cyanide complexes as highly anisotropic magnetic units. A fundamental characteristic of *4d*/*5d* cyanidometallates is the absence of single-ion magnetic anisotropy owing to their low spin ground state (*S* = 1/2) [21,22]. Hence, anisotropy is created in interplay with bounded high spin *3d* metal ions, through anisotropic exchange interactions [2,21,22]. The SCMs incorporating polycyanide metalloligands of heavier metals (4/5 *d*) are really less investigated [16,17,18,19,20]. The one dimensional (1D) coordination heterobimetallic polymers comprising low spin orbitally degenerate cyano complexes are particularly infrequent. There are only a few magnetic molecular wires based on octahedral hexacyanidometallates of Fe^III^ (*3d^5^*) [23,24,25,26], Os^III^ (*5d^5^*) [16], and three—involving pentagonal bipyramidal (PBP) heptacyanidomolibdate(III) (*4d^3^*) (Scheme 1a) [27,28].

The most studied are the magnetic materials based on PBP heptacyanidomolybdate(III) among which there are molecular assemblies of all dimensionalities; the Mn(II) complexes being high-spin partners for [Mo^III^(CN)_7_]^4−^ in such heterobimetallic complexes [8]. Among the members of this family there are 3D and 2D magnetic polymers with sufficiently high critical temperatures (above liquid nitrogen temperature of 77 K) [8]. The similar [Re(CN)_7_]^3−^ (isoelectronic analogue of [Mo^III^(CN)_7_]^4−^) compounds have been much less investigated. In addition, heterometallic compounds of heptacyanidomolybdate(III) and Mn(III) complexes are unknown, and their analogues for Re(IV) are only few.

**Scheme 2:**
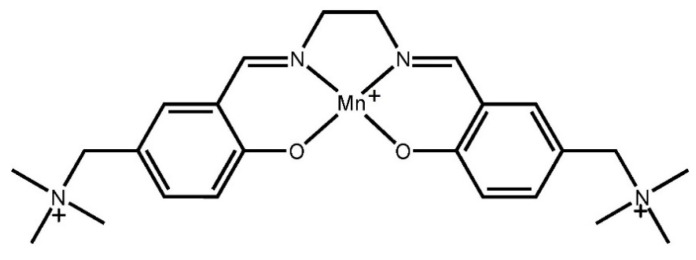
Molecular structure of tricationic *salen*-type [Mn(SB^2+^)]^3+^ high spin module.

The only SCM comprising PBP heptacyanidorhenate(IV) (*5d^3^*), being a neutral heterobimetallic polymer {[Mn^III^(SB^2+^)Re^IV^(CN)_7_]}_n_ (SB^2+^ is a dicationic Schiff base (SB) ligand, see Scheme 2), was communicated by us recently [29]. Our earlier attempts to prepare SMM or SCM by assembling trianion [Re^IV^(CN)_7_]^3−^ and monocationic Schiff base complex, [Mn^III^(acacen)]^+^ (acacen=N,N′-ethylenebis(acetylacetonylideneaminato), Scheme 1b) had resulted in a 3D framework {[Mn^III^(acacen)]_3_[Re^IV^(CN)_7_]}_n_ representing a material with strong magnetic anisotropy [30]. In addition, starting from the same compact cation [Mn^III^(acacen)]^+^ and an orbitally degenerate hexacyanido-metalloligand, we were able to obtain two 1D materials (Ph_4_P)_2_[Mn^III^(acacen)M^III^(CN)_6_] (M^III^ = Fe or Os) exhibiting slow magnetization relaxation and magnetic hysteresis [16,24]. Therefore, we had also expected to obtain the anionic molecular magnetic wires applying the large organic cations to compensate a negative charge of the {Mn^III^(acacen)Re^IV^(CN)_6_}^2–^ repeating unit in a chain. However, the using of the tetraphenylphosphonium (Ph_4_P) and bis(triphenylphosphine)iminium (PPN) as cations to led to a crystallization of two two-dimensional (2D) polymers, which are very different in magnetic behavior despite the same heterobimetallic layer composition.

In this paper, we present the preparation and investigation of the two layered materials: Ph_4_P[{Mn(acacen)}_2_Re(CN)_7_]·Solv (**1**) and PPN[{Mn(acacen)}_2_Re(CN)_7_]·Solv (**2**). These heterobimetallic compounds are the first representatives of 2D coordination polymers involving heptacyanidorhenate(IV), the magnetic network **2** possessing the record for molecular magnetic materials coercivity for cyanide-bounded molecular magnets.

## 2. Results and Discussion

### 2.1. Synthetic Approach

In the purposeful design of molecular magnetic materials, in addition to the optimal choice of initial molecular blocks (in our case, these are coordination compounds), synthetic chemists have to take into account many parameters for an appropriate self-assembling of paramagnetic units. A few words about our choice of the molecular precursors are needed. Theoretical calculations showed that among the orbitally degenerate cyanidometallates, the most promising are heptacyanide complexes with a pentagonal-pyramidal geometry of the coordination polyhedron [2,21] in contrast to octahedral hexacyanometallates. Since if even a slight deviation from the ideal geometry caused by crystal packing takes place, the complexes [M(CN)_7_]^n−^ retain their uniaxial symmetry, which plays a key role in ensuring magnetic anisotropy. In particular, orbitally degenerate heptacyanidorhenate(IV) (Scheme 1a) possesses a strong spin–orbit splitting (ζ_Re_ = 2400 cm^−1^) [31]. Further, its coordination polyhedron is much more rigid to preserve a pronounced uniaxial symmetry (D_5h_) in the heterobimetallic molecular magnetic materials, compared to its *4d* electronic analogue [Mo^III^(CN)_7_]^4−^. A partner for the heptacyanidorhenate must be a *d*-metal with a high spin ground state to guarantee strong magnetic exchange interactions and high critique temperatures [22]. Proceeding from the above and taking into account that Mn^II^-ion having maximal spin S = 5/2 for a *d*-metal reduces [Re^IV^(CN)_7_]^3−^ to [Re^III^(CN)_7_]^4−^ when assembled in solution under normal conditions [4], we therefore have chosen as a high-spin building unit, the Schiff base complex[Mn^III^(acacen)]^+^ (*3d^4^*), possessing the four unpaired electrons.

However, the reaction of (n-Bu_4_N)_3_[Re^IV^(CN)_7_] and [Mn^III^(acacen)Cl] with different ratios of reagents and independently of solvents (MeOH or CH_2_Cl_2_) resulted in very anisotropic hetero-bimetallic magnetic 3D material [{Mn^III^(acacen)}_3_Re^IV^(CN)_7_]_n_ (**3**) [30]. This is the only example of three-dimensional coordination polymer for the heterobimetallic assemblies involving homoleptic cyanides and Mn(III) Schiff base complexes. A salient feature of this neutral framework is the absence of solvate molecules, since there is no free space for them. The powder and crystal magnetic studies show that material **3** undergoes an antiferromagnetic ordering below a Neel temperature of 13 K.

Earlier, the use of tetraphenylphosphonium as a counterion resulted in the successful preparation of the 1D heterobimetallic anionic polymers (Ph_4_P)_2_[Mn^III^(acacen)M^III^(CN)_6_] (M^III^ = Fe or Os) with SCM properties [16,24]. This inspired us to keep probe the effectiveness of large organic cations in the synthesis of anionic low-dimensional molecular materials involving [Re^IV^(CN)_7_]^3−^ and [Mn^III^(acacen)]^+^ magnetic modules. However, when polymerization was carried out in an alcohol mixture (methanol, ethanol and isopropanol (1:1:1)) 2D material **1** was obtained for the first time. Furthermore, the replacement of Ph_4_P^+^ per bulkier PPN^+^ led to the formation of a 2D coordination network as well, instead of a chain polymer, giving compound **2** unique magnetic properties.

#### FT-IR Investigation

The FT-IR study data for **1** and **2** presented in Figures S1 and S2 (Supporting Information, SI) indicate the surprising similarity of these coordination polymers. The shapes and positions of the stretching vibration bands for cyanide ligands (ῡ(_CN_)) are very similar: 2127.4, 2090.8 and 2125.4, 2094.6 cm^−1^ for **1** and **2**, respectively. However, these values are significantly different from those for the precursor (Bu_4_N)_3_[Re(CN)_7_] (ῡ(CN)—2114 and 2074 cm^−1^) (Figure S3). This points out a bridging function of both apically and equatorially located CN groups. In the region of OH and CH vibrations, the IR spectra are also very similar. The main differences associated with the cationic parts of the compounds appear in the range 1700–400 cm^–1^.

### 2.2. Crystal Structure Description

**Figure 1:**
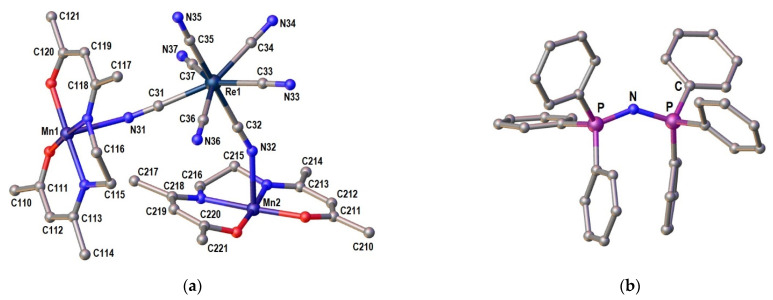
(**a**) Independent part, [{Mn(acacen)]_2_[Re(CN)_7_]^−^, of the anionic layer of **2** with the atom numbering scheme of the unique atoms; (**b**). Hydrogens atoms are omitted.

**Figure 2:**
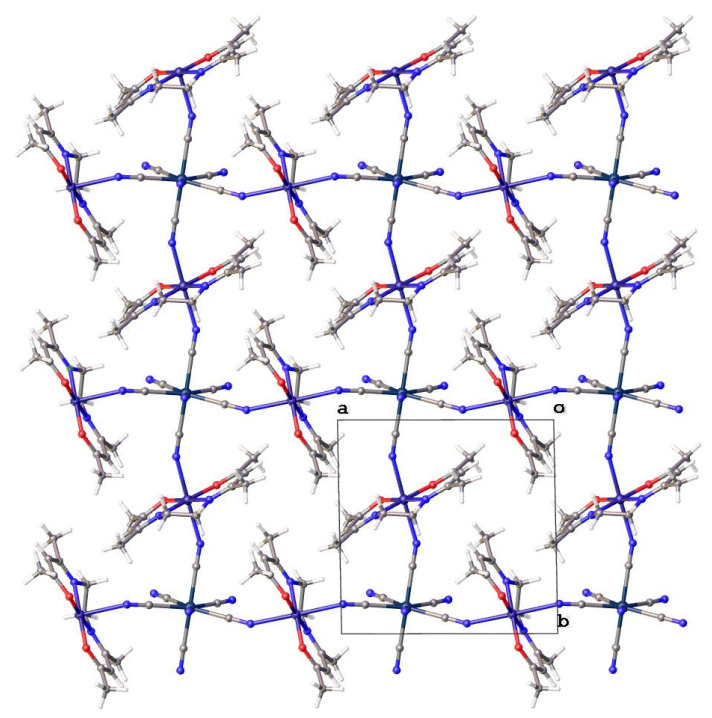
Projection of an anionic layer of **2** along the *c*-axis, showing a two-dimensional network structure consisting of two different crossed chains.

Although the single crystals were obtained for both substances, it was possible to solve the structure with a satisfactory R-factor only for compound **2**. The crystallographic data and structural refinement summary for **2** are included in Tables S1 (SI). Single crystal X-ray structural analysis revealed that compound **2** has a 2D molecular structure. The molecular views of the independent part, [{Mn(acacen)}_2_Re(CN)_7_]^−^, of the anionic layer of **2** and an organic cation PPN^+^ are shown in Figure 1. Figure 2 demonstrates a two-dimensional network structure of **2** consisting of two different crossed chains: in one, the [Re(CN)_7_]^3−^ metalloligands bind the neighboring [Mn(acacen)_2_]^+^ moieties with its apical axis Re and in the other, with the two equatorial CN ligands. Figures S4 and S5 (SI) show the hybrid 2D layered structure of **2** composed of anionic and cationic layers, presenting the view projected onto the *ac* and *bc* plane, respectively. The distances between the Re-centers located in the nodes of the 2D network correspond to the values of the crystal cell parameters *a*, *b*; while the distance between the planes of adjacent layers — *c*, parameters of 2D network of which (*a* = *b* = 10.9343(2) Å) being close to those of the neutral material [Mn(acacen)]_2_[Ru(CN)_5_NO] [32]. It should be noted that, at the moment, the 2D networks [{Mn(acacen)}_2_Re(CN)_7_]^–^ are the least symmetrical compounds compared to those of the previously studied [[K(18-cr)(2-PrOH)_2_] [Mn(acacen)]_2_[Fe(CN)_6_] [33] and K[Mn(acacen)]_2_[W(CN)_8_]·2H_2_O [34], the schematic representation of the anionic layers for them are presented in Figures S6 and S7.

Each [Re^IV^(CN)_7_]^3−^ anion coordinates to four neighboring trans-[Mn(acacen)]+ cations through four cyano nitrogens with the distances: Mn1–N31_apical_ of 2.384(3) Å and Mn2–N32_equat_ of 2.299(5) Å (for the selected geometric parameters of **2** see Table S2, SI,). The Mn–N≡C bond angle is 154.5(5) for apical cyanide and 155.2(3)° for the equatorial, pointing out that the CN ligands are tied to Mn(III) ions in a bent manner.

The Re coordination environment is close enough to a pentagonal bipyramid since Re-C distances are varying in 2.090(5)–2.115(3) Å range with the C—Re—C angles only slightly deviating from the ideal geometry (Table S2, SI). The geometry about Mn is a square plane bipyramide due to the Jahn–Teller effect of the high spin *d^4^* configuration of Mn^III^ and the strong in-plane donation of the *acacen*^2–^ ligand [33]. The Mn-to-ligand bond distances are in the 1.898(4)–1.988(6) Å range, whereas the axial Mn-N bond distances are elongated: Mn1—N31 = 2.384(3), Mn1—N34 = 2.321(3) Å and Mn2—N32 = 2.299(5), Mn2—N35 = 2.279(5) Å. Such a situation is typical for the hetero-bimetallic materials, including Mn^III^(SB) complexes and polycyanides metalloligands [35].

Space between the anionic layers is occupied by PPN^+^ cations and highly disordered solvent molecules, which were refined as water molecules when deposited in CCDC. However, in accordance with the result of elemental analysis, they were approximately localized as a mixture of water and ethanol in order to establish a system of hydrogen bonds. It should be especially noted that the crystals of **2** are prone to partially lose the solvent retaining their integrity.

Although several crystals of **1** were examined, unfortunately, their structure was only partially resolved. The SCXRD experimental details for **1** are summarized in Table S3 (SI). It should be noted that the found cell parameters for **1** (*a* = *b* = 15.1259 (15), *c* = 61.625 (7) Å, β =90°, *V* = 14105 (3) Å^3^), *Z =* 8) are much larger than those for **2**. However, a layered structure was also established for **1**: four layers per cell are located perpendicularly to the *c* axis (Figures S8–S11, SI).

**Figure 3:**
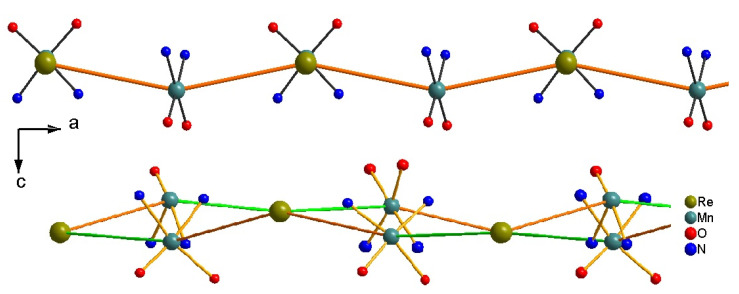
Schematic presentation of the anionic layer of **2** (top) and **1** (bottom), projection along the *b*-axis. The Re-CN_apical_—{MnN_2_O_2_} and Re-CN_equatorial_—{MnN_2_O_2_} connections are colored in green and orange, respectively.

The space group was chosen based on a distribution of heavy atoms. In addition, we were able to localize most of the atoms from the nearest environment of Re and Mn. This was sufficient to establish an organization of the layers. Note that in the 2D network of **2**, there are two types of perpendicular chains in one of which the Mn ions are connected by two apical cyanides of the metalloligand and in the other—by equatorial CN-groups. While in material **1**, the layers are more “corrugated” (Figure 3), owing to their mutually perpendicular chains being organized equally: the Mn centers are connected to the Re ions both by apical and equatorial cyanides (Figures S9 and S10, SI). This is manifested in the distances between metal centers. If in a layer of **2**, there are only two types of distances between Mn atoms 10.8268(5) and 1.7016(5) Å (for apical and equatorial cyanides respectively), then in the layers of **1**, there are as many as four possible distances Mn–Mn for each layer (for a middle layer, the “apical” distances are 10.7825(8), 10.7612(8) Å; “equatorial”—10.6374(5), 10.6173(5) Å). These structural features are extremely important for understanding the difference in magnetic behavior of **1** and **2**.

### 2.3. Magnetic Behavior Investigation

#### 2.3.1. Static Magnetic Studies

**Figure 4:**
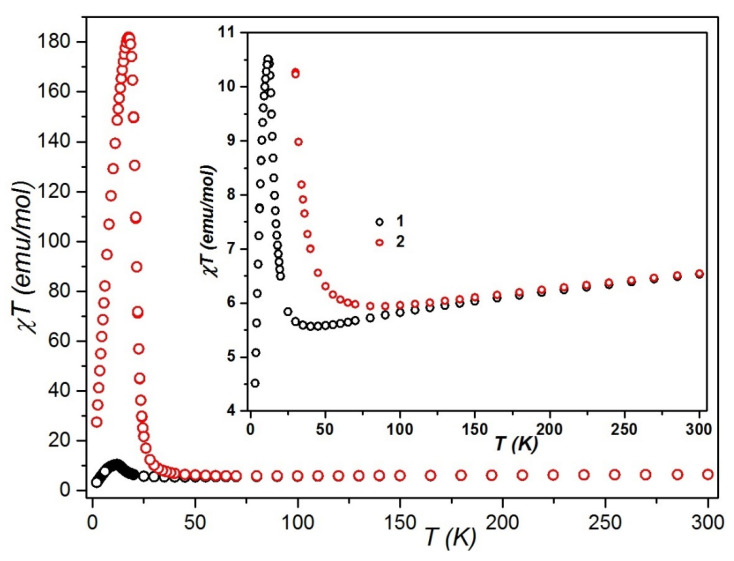
Magnetic *dc* plots for **1** and **2**. Temperature dependencies of *χT* at H = 1000 Oe. Inset: zooms magnetization versus the field at *T* = 2 K.

The temperature dependencies of the *dc* molar susceptibility for the powder samples of **2** and **1,** measured in an applied field of 1000 Oe, are shown in Figure 4 as *χT vs T* plots. At 300 K, the observed *χT* value of 6.55 and 6.50 emu K mol^−1^ (for **2** and **1**, respectively) are close to 6.51 emu K mol^−1^ expected for two Mn^III^ (S = 2, g = 2.03) [30] and one Re^IV^ (S = 1/2, g = 2.33) [36] as magnetically uncoupled spin carriers. As can be seen in the inset of Figure 4, which zooms the *χT–T* plots in the *Y* axis from 4 to 11 emu K mol^−1^, the magnetic behavior of both materials is very similar. This is especially true for the high temperature region. Starting from room temperature, *χT* curves first decrease slightly and then reach a light minimum of 5.94 emu K mol^−1^ at ~85 K for **1** and 5.57 emu K mol^−1^ at ~85 K for **2**. With a further decrease in temperature, *χT* plots increase and reach maximums of 10.51 emu·K·mol^−1^ at ~11.5 K for **1** and 181.45 emu·K·mol^−1^ at 18 K for **2**, before dropping to 7.75 and 27.54 emu K mol^−1^ at 2 K for **1** and **2**, respectively. This behavior of *χT* is typical of ferromagnetic coupling between the Mn^III^ and Re^IV^ ions within the chains [29,30].

**Figure 5:**
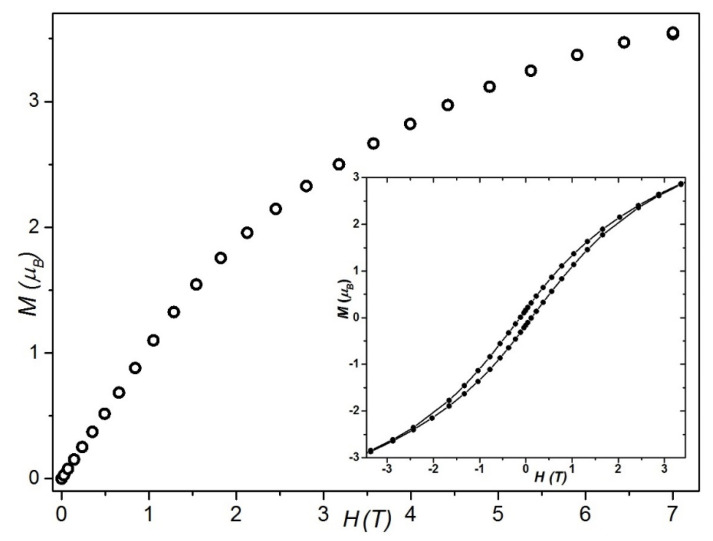
Initial magnetization *vs* field plot and magnetic hysteresis (inset) for **1** at *T* = 1.8 K.

**Figure 6:**
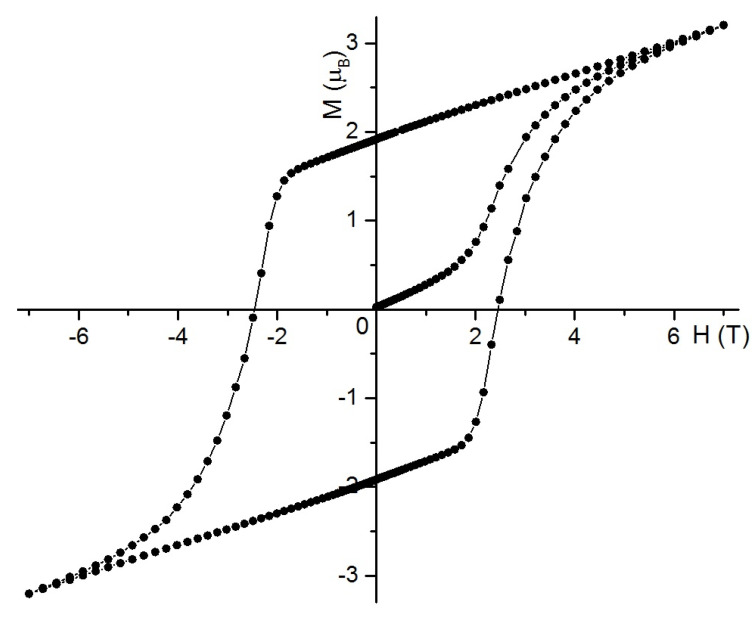
Initial magnetization *vs* the field plot and magnetic hysteresis for **2** at *T* = 1.8 K.

The M(H) plots for **2** and **1** are shown in Figure 5 and Figure 6, respectively. It should be noted that the value of M of 3.62 *μ_B_* at 7 T for **1** is close to the 3.58 *μ_B_* found for the neutral SCM {[Mn^III^(SB^2+^)Re^IV^(CN)_7_]}_n_ [29]. For **2**, the initial magnetization curve for **2** has an S-shape characteristic for metamagnets, indicating that at 2 K a metamagnetic transition occurs in the field of 23205 Oe, the value extracted from the *dM/dH* plot (Figure S13). The M value of 3.2 *μ_B_* at the maximum available field of 7 T is very far from 7 *μ_B_* calculated for antiferromagnetically coupled Mn^III^ (*S* = 2) and Re^IV^ (*S* = ½) with a *g_av_* = 2.0. Note that the estimated by a linear approximation limit of anisotropic magnetic field *H_A_* for **2** is approximately 26 T. This is significantly greater than the value of 15 T found for the {[Mn^III^(SB^2+^)Re^IV^(CN)_7_]}_n_ chain [29]. Studies of initial M(H) plots for **2** at different temperatures (Figure S14) show that the S-shape of the curve disappears at the temperatures above 5 K. Such a behavior of magnetization below 5 K is reminiscent of a metamagnetic transition from an antiferromagnetic state to a polarized, similar to that found for the related 3D material **3** [30].

As can be seen in Figure 5, Figure 6 and Figure S15, both materials possess a magnetic hysteresis. At 1.8 K, compound **1** shows a hysteresis loop with a small coercive field of 1150 Oe, while for **2** this parameter reaches a value of 2.486 T, which is much greater than that of 1.5 T observed for the SCM [Mn^II^(bida)(H_2_O)]_2_[Mo^III^(CN)_7_]·6H_2_O [19]. The M(H) of **2** does not saturate even in the field of 7 T (70 kOe) and exhibits a linear dependence at high fields, which is characteristic for strongly anisotropic magnetic materials. The magnetic hysteresis plots of **2** measured at higher temperatures are presented in Figure S15. Inset of this figure demonstrates that a very tiny hysteresis loop is still visible at 16 K, indicating that a critical temperate for this material is only slightly higher than 16 K.

#### 2.3.2. Dynamic Magnetic Studies

To probe the magnetic dynamics of 2D networks [Mn^III^(acacen)Re^IV^(CN)_7_]^2−^, the temperature and frequency dependent *ac* susceptibilities data were collected. The intriguing results of this study are presented in Figures S16 and S17 for **1** and **2**, respectively.

Unfortunately, the dynamic studies have not been carried out at zero dc field. For this reason, field induced behavior for both is presented. The variable temperature of the in-phase (*χ*’) and out-of-phase (*χ*”) parts of *ac* molar susceptibilities for both compounds were studied under H_dc_ = 100 Oe, H_ac_ = 3 Oe. Both *ac* components of **1** are frequency dependent (Figure S16). The *χ*’ susceptibility of **1** displays apparently the two relaxation processes, while no second relaxation process was found for *χ*”.

For a magnetically anisotropic chain compound, such as the ordinarily studied anisotropic Heisenberg or Ising model chains, *χ*′*T* is proportional to the correlation length *ξ*, and follows the equation *χT* ≈ *C*_eff_exp(Δ_ξ_/*k*_B_*T*) where *C*_eff_ is the effective Curie constant per magnetic unit and Δ_ξ_ is the energy required to create a domain wall [4,5]. Therefore, to determine the single-chain magnet behavior of a material, the ln(*χ*′*T*) *vs* 1/*T* plot has to be investigated in detail. Its linear character, with a slope corresponding to Δ*_ξ_*, attests to the SCM nature of the system and the manifestation of significant magnetic anisotropy.

The respective ln(*χ*′*T*) *vs* 1/*T* plot for **1** is presented in Figure S18 (SI). A linear region is detected in the range of 11–8.5 K, giving *C_eff_* = 0.48 emu^ ^K mol^−1^ and Δξ/k_B_ = 3.62 K. At 8 K, ln(*χ′T*) reaches a maximum and then displays a linear drop with lowering of temperature until *χ*′ is blocked under a field oscillation of 0.56 Hz. A crossing of the two linear parts occurs at ca. 7.1 K, representing a crossover temperature T* [37]. Unfortunately, the hysteresis loop for **1** was registered only at the temperature of 1.8 K, and it is not possible to extract the magnetization blocking temperature (T_B_) for **1**. However, it can be assumed that the dependence of *χ*′ on frequency is due to slow magnetic relaxation of the chains, from which the 2D network of **1** is composed.

A similar analysis carried out for **2** is shown in Figure S19. A linear region is perceived in the range of 24–21.5 K with Δξ/k_B_ = 326.74 and *C_eff_* = 11.42 emu^ ^K mol^−1^. The crossover temperature, T* = 20.1 K is rather close to T_B_ of ~16 K found from the magnetization measurements for **2**. In addition, the insufficient amount of registered points for the *ac* susceptibility, makes it very difficult to determine both the *χ*′’ maxima for both compounds and the *χ*′ maxima for **2**. For the latter, however, the position of these maxima can be roughly estimated as 19.75 K (Figure S19, SI), a value very close to T*. Unlike the studied 2D networks [Mn^III^(acacen)Re^IV^(CN)_7_]^2−^ in this paper, the frequency dependence in the *ac* data was not found for their “next of kin”—3D framework **3** [30]. However, for the latter a sharp phase transition was observed at Neel temperature, T_N_ = 13 K.

The above data of the combined *dc* and *ac* magnetic studies, as well as the positive results of testing on SCM behavior for both compounds, indicate the presence of magnetization relaxation in the chains forming the anion layers of coordination polymers **1** and **2**. As it follows from the single crystal X-ray diffraction data, these layers are quite well isolated from each other by organic cations, which prevent the formation of a pseudo 3D structure. Consequently, the magnetic hysteresis recorded for these two materials is scarcely the result of a phase transition, but is due to the presence of mutually perpendicular 1D polymers, which are composed of anisotropically coupled spin carriers. This is confirmed by a significant difference in the magnetic behavior of **1** and **2**, since there is a fundamental dissimilarity in the structure of the Re-Mn-Re-Mn chains for these materials. The much lower T_B_ for **1** is due to the alternating binding of Mn-ions by the metalloligand [Re(CN)_7_]^3–^ into the chain: either through apically located cyanides or through the equatorial. Moreover, such alternating binding is the same for both groups of perpendicularly located 1D fragments of the layer. Whereas in **2**, these 1D fragments differ significantly in the manner of binding—apical in one type of the parallel chains and equatorial in the chains perpendicular to the former. This combination considerably increases the magnetic anisotropy of the Re(IV)-Mn(III) heterobimetallic system, highlighting the direction along the crystallographic axis *b* as a direction of easy magnetization. Whereas the orientation along *a* most likely gives the smaller magnetization, and the orientation along *c* should match to the hard magnetization axis.

Of particular note is the coercive field of 2.486 T for **2**, which is a record for heterobimetallic molecular magnets, including homoleptic polycyanide metalloligands. However, the coercivity of **2** also exceeds those of 1.375 T found for a 2D metal-organic network Li_0.7_[Cr^II^(pyz^•−^)_2_]Cl_0.7_·(THF)] [38], and only 1D {Co-NIT}_n_ (NIT—nitronyl nitroxide radical) magnetic wires are superior in coercivity to complex 2 [39].

The unusual magnetic properties of both compounds originate from the interplay of Re−Mn anisotropic spin coupling and the ZFS effect of Mn^III^ ions with a noncollinear orientation of the local magnetic axes in crystals of the compounds. Both materials possess magnetic anisotropy. It is especially strong for compound **2**, which is confirmed by the extremely high values of coercivity and anisotropy field H_A_.

## 3. Perspectives

Since no similar 2D networks with SCM behavior and magnetic hysteresis have been obtained so far, theoretical modeling of magnetic properties of the two-dimensional systems based on anisotropically coupled molecular blocks is a challenge. Therefore, we have to perform additional experiments to study the dynamic properties at temperatures below 2 K for both material **2** and material **1**. In addition, for the latter, it is necessary to investigate the magnetic hysteresis on an oriented single crystal at different temperatures in the range from 30 mK to 7 K. Magnetic measurements on an oriented crystal in the three different directions (relative to a direction of the applied field H) are also necessary for material **2,** since this will help us to obtain specific information about the influence of different binding types in 1D fragments of the layer on anisotropy of the system.

We invite fellow theoreticians to participate in this project on modeling magnetic behavior oh these highly anisotropic systems. In addition, we are also open to an offer from crystallochemists to assist in the complete deciphering of the structure for compound **1**.

## 4. Materials and Methods

All of the reagents and solvents (EKOS-1, Moscow, Russia) were used without further purification; [Mn(SB^2+^)(H_2_O)_2_](ClO_4_)_3_·H_2_O [40] and (Bu_4_N)_3_[Re(CN)_7_] [36] were prepared using published protocols.

The polycrystalline powder material of {[Mn(SB^2+^)Re(CN)_7_]·7H_2_O}_n_ (**1**) was obtained via a precipitation method using a 1:3 mixture of water and acetonitrile as the solvent.

*Synthesis of **1***. The solids (n-Bu_4_N)_3_Re(CN)_7_ (175 mg, 0.16 mmol), Mn(acacen)Cl (50 mg, 0.16 mmol) and Ph_4_PCl (120 mg, 0.32 mmol) were placed in a vial and filled with 3 mL of a mixture of alcohols (MeOH: EtOH: i-PrOH = 1:1:1). The reaction mixture was stirred for a short time with gentle heating, filtered through a piece of cotton. The latter was rinsed with 0.5 mL of the same alcohol mixture, and this filtrate was added to the main solution, which was closed with a holey parafilm and left for three days in a dark place. Dark flat-square crystals were filtered off, washed with a small amount of isopropanol and dried in air. The yield was 85% (Mn based). C_55_H_56_Mn_2_N_11_O_4_PRe(H_2_O)_2_(EtOH)_2_ (1390.33); CHN: calcd. C 50.97, H 5.22, N 11.08; found C 50.9, H 5.2, N 11.0. IR (KBr) ῡ(_CN_): 2127.4, 2090.8 cm^−1^.

*Synthesis of **2***. Preparation method is similar to those of **1** with using PPNCl (185 mg, 0.35 mmol). The yield was 87% (Mn based). C_67_H_66_Mn_2_N_12_O_4_P_2_Re·H_2_O·C_2_H_5_OH (1525.43); CHN: calcd. C 54.33, H 4.89, N 11.02; found C 54.2, H 5.1, N 11.0IR (KBr) ῡ(_CN_): 2125.4, 2094.6 cm^−1^.

Elemental (C,H,N) analysis was carried out with a Euro-Vector 3000 analyzer (Eurovector, Redavalle, Italy). FTIR spectra were recorded using Scimitar FTS 2000 spectrophotometer (Digilab LLC, Canton, MA, USA) (KBr pellets) in 4000–375 cm^−1^ range. Powder X-ray measurements were performed using Cu-Kα radiation (λ = 1.5418 Å) with an X’Pro powder diffractometer (PANalytical Inc., Almelo, The Netherlands) at room temperature. All magnetic measurements of properties were performed using a Quantum Design Quantum Design MPMS^®^3 magnetometer (Quantum Design, Inc., San Diego, CA, USA) in the temperature range of 1.8–300 K and under a magnetic field of up to 70 kOe. The magnetic susceptibility *χ* is the molar magnetic susceptibility per mole of **1** and **2** units and was corrected for the diamagnetic contribution calculated from Pascal’s constants [41].

Single-crystal XRD experimental details are presented in Table S1 (Supplementary Materials).

The solvents were assigned to disordered water molecules. However, their positions can be partially occupied by the molecules of alcohols, which were used as a solvent.

## Data Availability

The complete crystallographic data for **2** have been deposited with the Cambridge Crystallographic Data Centre under the reference numbers CCDC 1566470. These data can be obtained, free of charge, from CCDC via www.ccdc.cam.ac.uk/structures (accessed on 29 October 2022).

## Supplementary Materials

The following supporting information can be downloaded at: https://www.mdpi.com/article/10.3390/ma15238324/s1, Figure S1: FTR-IR spectrum for {Ph_4_P[Mn(acacen)Re(CN)_7_]·Solv}_n_ **1**; Figure S2: FTR-IR spectrum for {PPN[Mn(acacen)Re(CN)_7_]·Solv}_n_ **2**; Figure S3: IR spectrum for the precursor, (Bu_4_N)_3_[Re(CN)_7_]·H_2_O (KBr); Table S1: SCXRD Experimental details for **2**; Figure S4: Projections of hybrid layered structure of **2** onto the *ac* plane; Figure S5: Projections of hybrid layered structure of **2** onto the *bc* plane; Table S2: Selected geometric parameters for **1**; Figure S6: A layer formation in a crystal of [K(18-cr)(2-PrOH)_2_][{Mn(acacen)}_2_Fe(CN)_6_]; Figure S7: A layer formation in a crystal of K[Mn(acacen)]_2_[W(CN)_8_]·2H_2_O. The acacen^2-^-ligands are reduced, and hydrogen atoms are omitted for clarity; Table S3: SCXRD Experimental details for **1**; Figure S8: A view of four layers in **2** along *c*—axis. The connections Re-CN_apical_—{MnN_2_O_2_} and Re-CN_equatorial_—{MnN_2_O_2_} are colored in green and orange respectively; Figure S9: A view of four layers in **2** along *a*—axis. The connections Re-CN_apical_—{MnN_2_O_2_} and Re-CN_equatorial_—{MnN_2_O_2_} are colored in green and orange respectively; Figure S10: A view of four layers in **2** towards plane hkl = 123. The connections Re-CN_apical_—{MnN_2_O_2_} and; A view of superimposed in pairs layers in for **1**,: the first on the second, the second on the third and the third on the fourth Re-CN_equatorial_—{MnN_2_O_2_} are colored in olive and red respectively; Figure S11: A view of superimposed in pairs layers in for **1**: the first on the second, the second on the third and the third on the fourth; Figure S12: PXRD pattern for the material **1**; Figure S13: *dM/dH* plot of **2** for the magnetization data collected at 1.8 K; Figure S14: Initial magnetization plots of **2** measured at different temperatures on a polycrystalline sample; Figure S15: Magnetic hysteresis plots of **2** measured at different temperatures. Inset is a zoom of low field region for the data collected at 10 and 16 K; Figure S16: Variable-temperature of the real, *χ*’ (top), and imaginary, *χ*” (bottom), parts *ac* molar susceptibility data for **1** under H_dc_ = 100 Oe, H_ac_ = 3 Oe. Solid lines are guides; Figure S17: Variable-temperature of the real, *χ*’ (top), and imaginary, *χ*” (bottom), parts *ac* molar susceptibility data for **2** under H_dc_ = 100 Oe, H_ac_ = 3 Oe. Solid lines are guides; Figure S18: Plots of ln(X′*T*) vs 1/*T* (where X′ is a real component of the *ac* susceptibility for **1** collected in applied *dc* field of 100 Oe, H_ac_ = 3Oe and frequency of 0.56 Hz. The dashed lines correspond to a linear fit.; Figure S19: Plots of ln(*χ*′T) vs 1/*T* (where *χ*′ is a real component of the *ac* susceptibility for **2** collected in applied *dc* field of 100 Oe, H_ac_ = 3Oe and frequency of 0.56 Hz. The dashed lines correspond to a linear fit.
