# Supplementary material for: Very Anisotropic 2D Molecular Magnetic Materials Based on Pentagonal Bipyramidal Heptacyanidorhenate(IV)
Source: Materials (Basel). 2022 Nov 23;15(23):8324. doi: 10.3390/ma15238324 (PMC9739847; doi:10.3390/ma15238324)
Supplement: Supplementary file 1 [file materials-15-08324-s001.zip › materials-2033067-supplementary.pdf]

Supporting Information

Very Anisotropic 2D Molecular Magnetic Materials Based on Pentagonal Bipyramidal Heptacyanidorhenate(IV)

Eufemio Moreno Pineda 1, Wolfgang Wernsdorfer 2 and Kira E. Vostrikova 3,*

[1] Nikolaev Institute of Inorganic Chemistry SB RAS, 3 Lavrentiev Avenue, 630090 Novosibirsk, Russia;
[2] Physikalisches Institut, Karlsruhe Institute of Technology, 1 Wolfgang-Gaede-Str., D-76131 Karlsruhe;
[3] Departamento de Física, Facultad de Ciencias Naturales, Exactas y Tecnología, Universidad de Panamá, 0874, Panamá

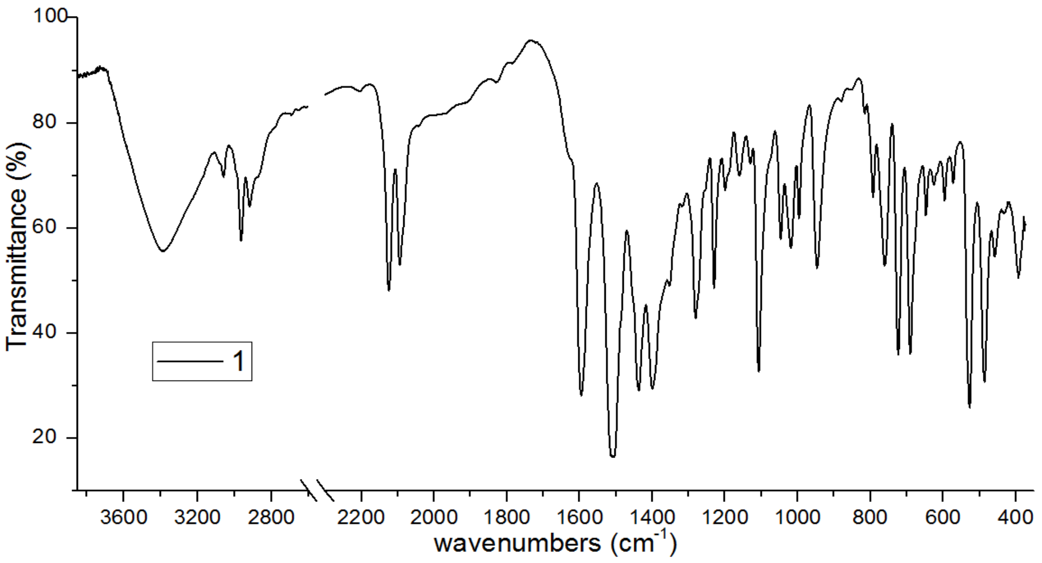

Figure S1. FTR-IR spectrum for $\{Ph_4P[Mn(acacen)Re(CN)_7]\cdot Solv\}_n$ **1**.

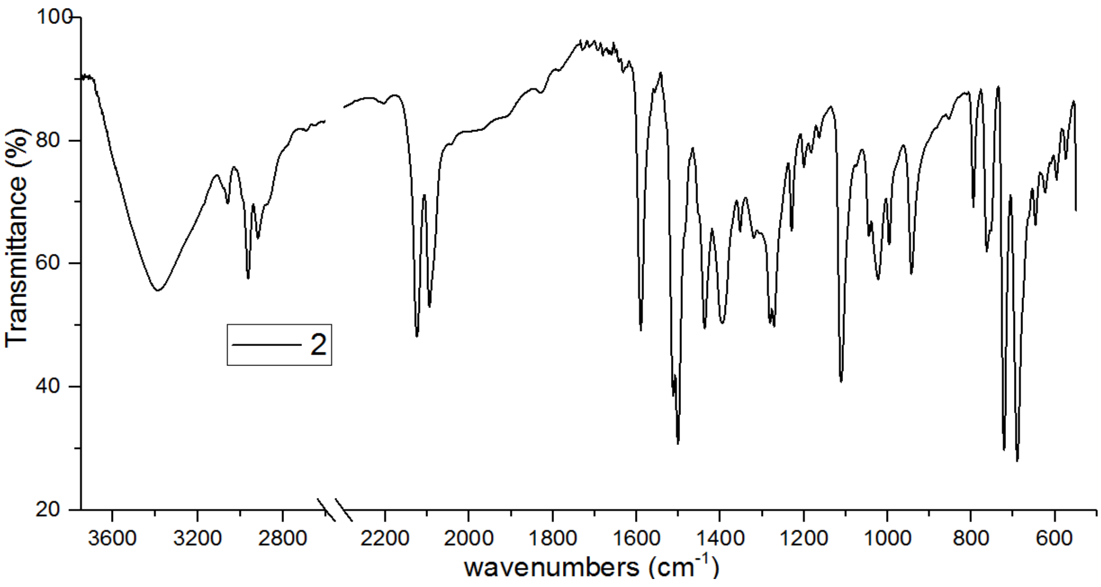

Figure S2. FTR-IR spectrum for $\{PPN[Mn(acacen)Re(CN)_7]\cdot Solv\}_n$ **2**.

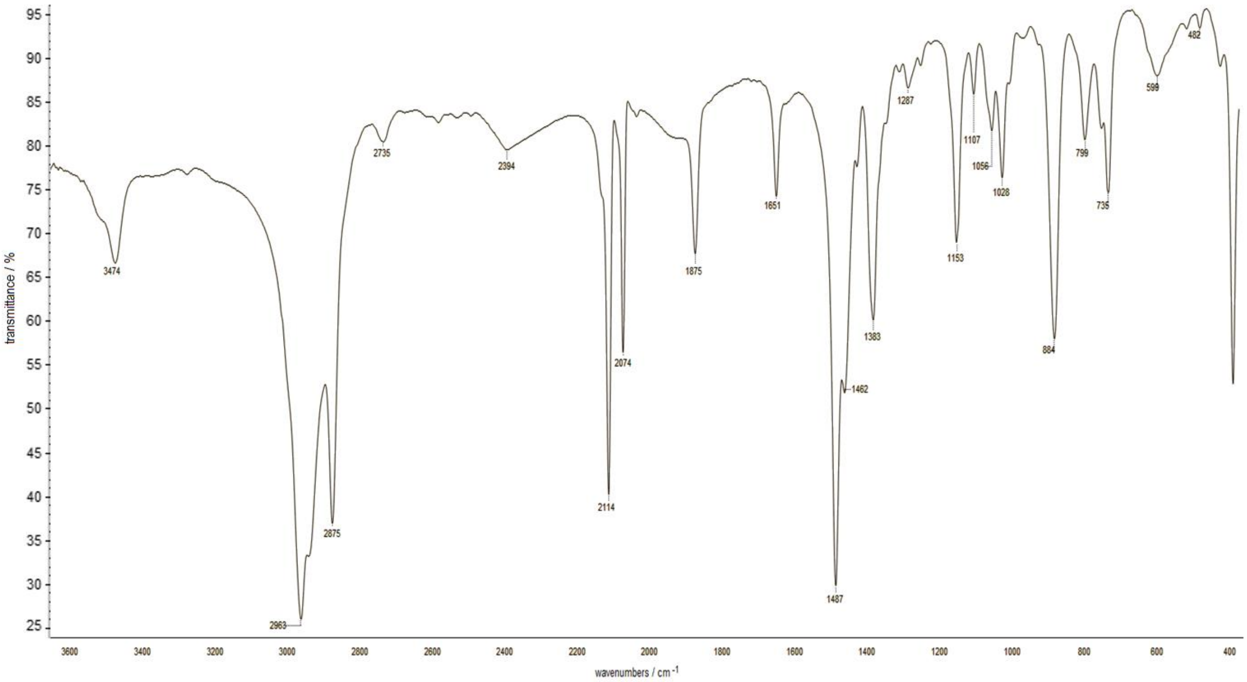

Figure S3. IR spectrum for the precursor, $(Bu_4N)_3[Re(CN)_7]\cdot H_2O$ (KBr).

Table S1. SCXRD Experimental details for **2**.

Crystal data for **2**	
Chemical formula	$C_{31}H_{36}Mn_2N_{11}O_4Re\cdot C_{36}H_{30}NP_2\cdot 3(H_2O)$
M_r	1515.39
Crystal system, space group	Triclinic, *P*1
Temperature (K)	298
a, *b*, *c* (Å)	10.7016 (5), 10.8268 (5), 16.2004 (7)
α, β, γ (°)	102.662 (1), 93.607 (1), 90.207 (1)
V ($Å^3$), *Z*	1827.47 (14), 1
Radiation type<br>μ (mm^{-1})	Mo *K*α<br>2.09
Crystal size (mm)	0.25 × 0.25 × 0.1
Data collection	
Diffractometer	CCD area detector diffractometer Bruker X8 APEX
Absorption correction: Multi-scan: *SADABS2012*/1 (Bruker, 2012) was used for absorption correction. wR_2 (int) was 0.0717 before and 0.0572 after correction. The Ratio of minimum to maximum transmission is 0.9183. The λ/2 correction factor is 0.0015.	
T_{min}, T_{max}	0.685, 0.746
No. of measured, independent and observed [$I > 2\sigma(I)$] reflections 27331, 14844, 14841	
R_{int}<br>$(\sin \theta/\lambda)_{max}$ ($Å^{-1}$)	0.019<br>0.679
Refinement	
$R[F^2 > 2\sigma(F^2)]$, $wR(F^2)$, S	0.020, 0.051, 1.08
No. of reflections<br>No. of parameters<br>No. of restraints	14844<br>817<br>6
H-atom treatment	H-atom parameters constrained
$\Delta\rangle_{max}$, $\Delta\rangle_{min}$ (e $Å^{-3}$)	1.38, -0.35
Absolute structure: Flack x determined using 5300 quotients [(I+)-(I-)]/[(I+)+(I-)] (Parsons, Flack and Wagner, Acta Cryst. B69 (2013) 249-259).	
Absolute structure parameter	0.017 (2)

Absorption corrections were applied with the use of the SADABS program [APEX2 (Version 2.0), SAINT (Version 8.18c), and SADABS (Version 2.11), Bruker Advanced X-ray Solutions, Bruker AXS Inc., Madison, Wisconsin, USA, 2000–2012.]. The crystal structure was solved by direct methods and refined by full-matrix least squares techniques with the use of the SHELXTL package [Sheldrick, G.M. (2015). Acta Cryst. C71, 3-8]. Computer programs: *APEX3* (Bruker-AXS, 2016), *SAINT* (Bruker-AXS, 2016), *SHELXT* 2014/5 (Sheldrick, 2014), *SHELXL2017*/1 (Sheldrick, 2017). Atomic thermal displacement parameters for non-hydrogen atoms were refined anisotropically. The positions of hydrogen atoms were calculated corresponding to their geometrical conditions and refined using the riding model.

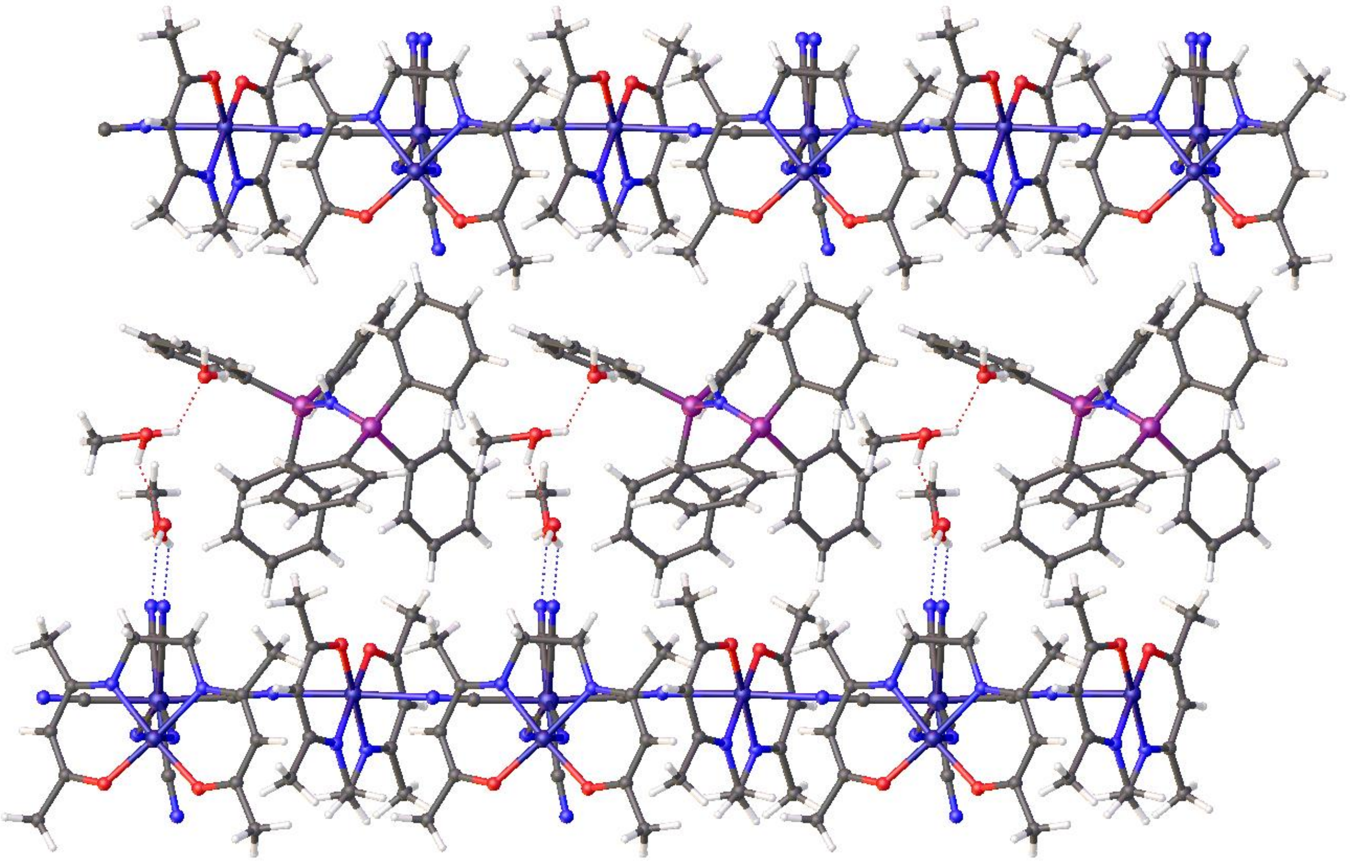

Figure S4. Projections of hybrid layered structure of **2** onto the *ac* plane.

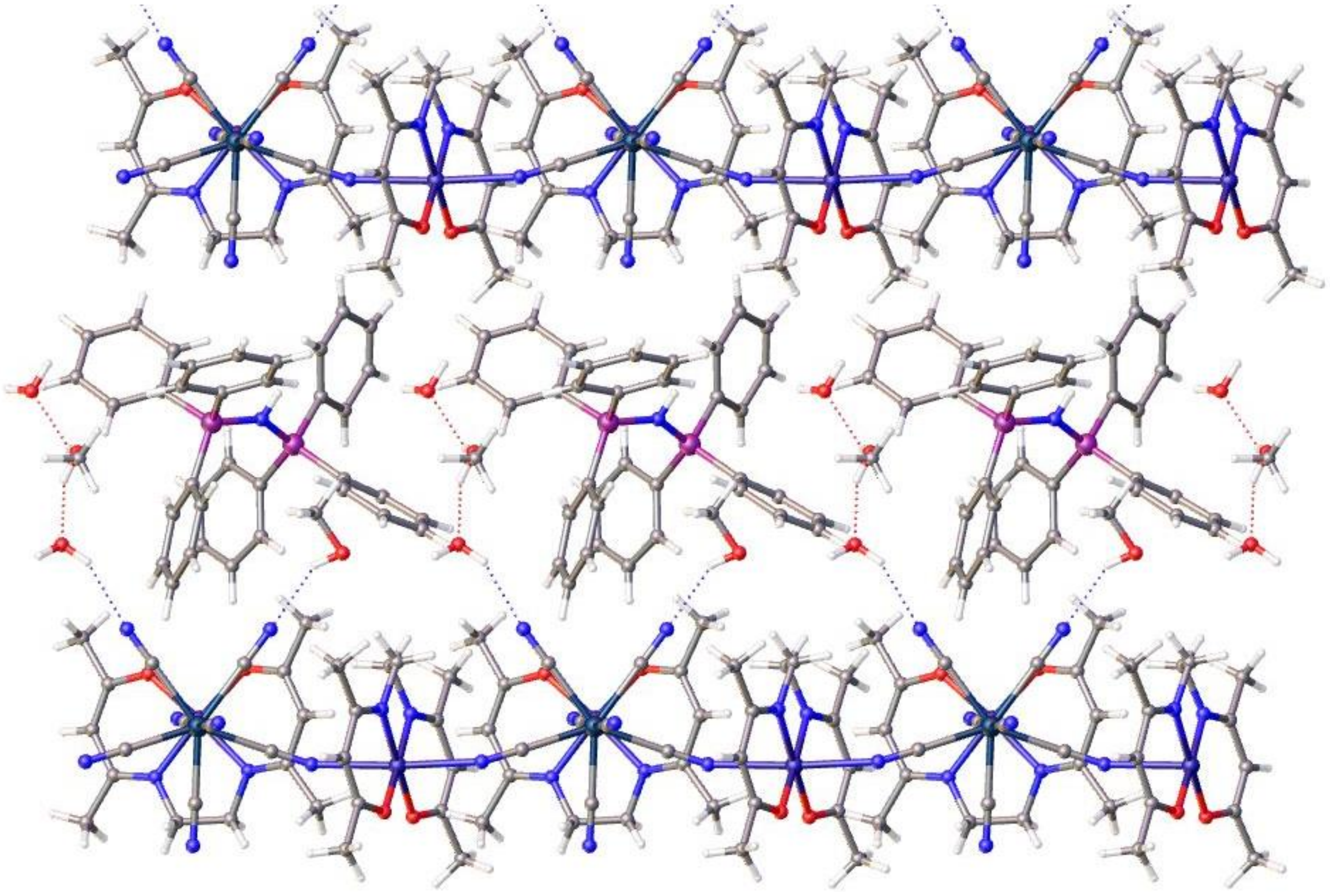

Figure S5. Projections of hybrid layered structure of **2** onto the *bc* plane.

Table S2. Selected geometric parameters for **1**.

Bond	Bond length, Å	Bond	Bond length, Å
Re1—C31	2.115(3)	Mn2—O21	1.898(4)
Re1—C32	2.090(5)	Mn2—O22	1.901(4)
Re1—C33	2.113(4)	Mn2—N21	1.973(6)
Re1—C34	2.096(3)	Mn2—N22	1.988(6)
Re1—C35	2.104(5)	Mn2—N32	2.299(5)
Re1—C36	2.108(4)	Mn2—N35	2.279(5)
Re1—C37	2.108(5)	N31—C31	1.140(5)
Mn1—O11	1.908(3)	N32—C32	1.155(6)
Mn1—O12	1.899(3)	N33—C33	1.144(6)
Mn1—N11	1.974(3)	N34—C34	1.155(5)
Mn1—N12	1.967(4)	N35—C35	1.136(7)
Mn1—N31	2.384(3)	N36—C36	1.149(5)
Mn1—N34	2.321(3)	N37—C37	1.154(8)

Bond angle	Angle (°)	Bond angle	Angle (°)
C32—Re1—C35	176.2(3)	N31—C31—Re1	177.4(3)
C34—Re1—C33	71.11(15)	N32—C32—Re1	178.1(5)
C34—Re1—C37	72.20(16)	N33—C33—Re1	178.1(4)
C36—Re1—C31	72.16(14)	N34—C34—Re1	179.1(3)
C36—Re1—C33	73.00(15)	N35—C35—Re1	177.4(6)
C37—Re1—C31	72.37(15)	N36—C36—Re1	178.5(4)
C31—N31—Mn1	155.2(3)	N37—C37—Re1	175.6(7)
C32—N32—Mn2	154.5(5)		

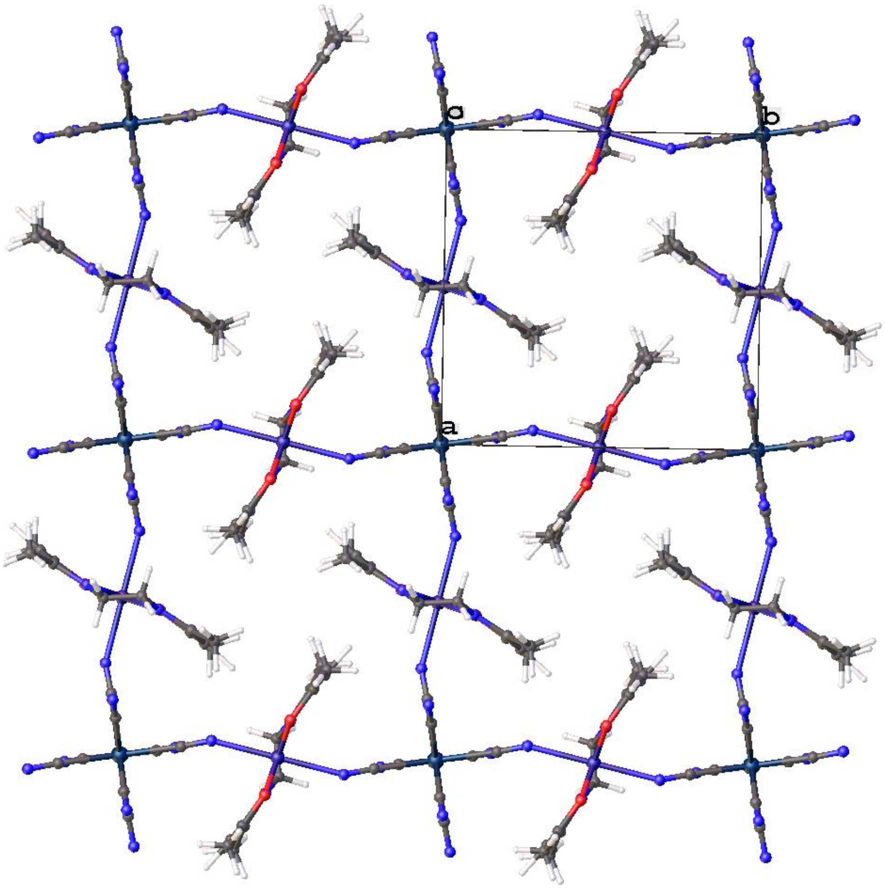

Figure S6. A layer formation in a crystal of [K(18-cr)(2-PrOH)$_2$][{Mn(acacen)}$_2$Fe(CN)$_6$] (10.1021/ic980448p).

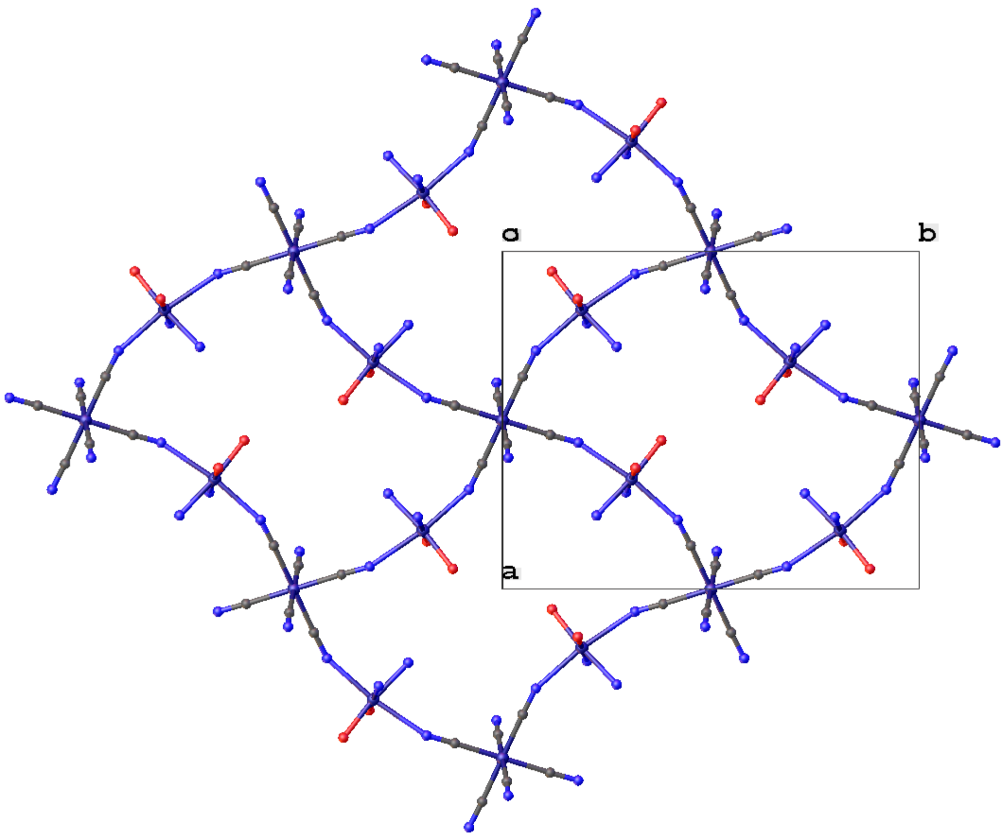

Figure S7. A layer formation in a crystal of K[Mn(acacen)]$_2$[W(CN)$_8$]·2H_2O. The acacen^{2-}-ligands are reduced, and hydrogen atoms are omitted for clarity. (10.1016/j.inoche.2004.08.017)

Table S3. SCXRD Experimental details for **1**.

Crystal data	
Chemical formula, M_r	$C_{70}Mn_2N_{12}O_7P_2Re$, 1478.9
Crystal system, space group	Monoclinic, $P112_1$
Temperature (K)	293
a, b, c (Å)<br>β (°)<br>V (Å^3), Z	15.1259 (15), 15.1318 (16), 61.625 (7)<br>90<br>14105 (3), 8
Radiation type<br>μ (mm^{-1})	Mo Kα<br>2.17
Data collection	
Diffractometer	Bruker CCD diffractometer
Absorption correction	For a sphere Jana2006
T_{min}, T_{max}	0.722, 0.727
No. of measured, independent and observed [$I > 3\sigma(I)$] reflections	35290, 23617, 22136
R_{int}<br>$(\sin \theta/\lambda)_{max}$ (Å^{-1})	0.030<br>0.606
Refinement	
$R[F^2 > 2\sigma(F^2)]$, $wR(F^2)$, S	0.146, 0.213, 5.35
No. of reflections<br>No. of parameters	23617<br>386
$(\Delta/\sigma)_{max}$<br>$\Delta\rho_{max}$, $\Delta\rho_{min}$ (e Å^{-3})	3.060<br>9.07, −3.20
Absolute structure	8638 of Friedel pairs used in the refinement

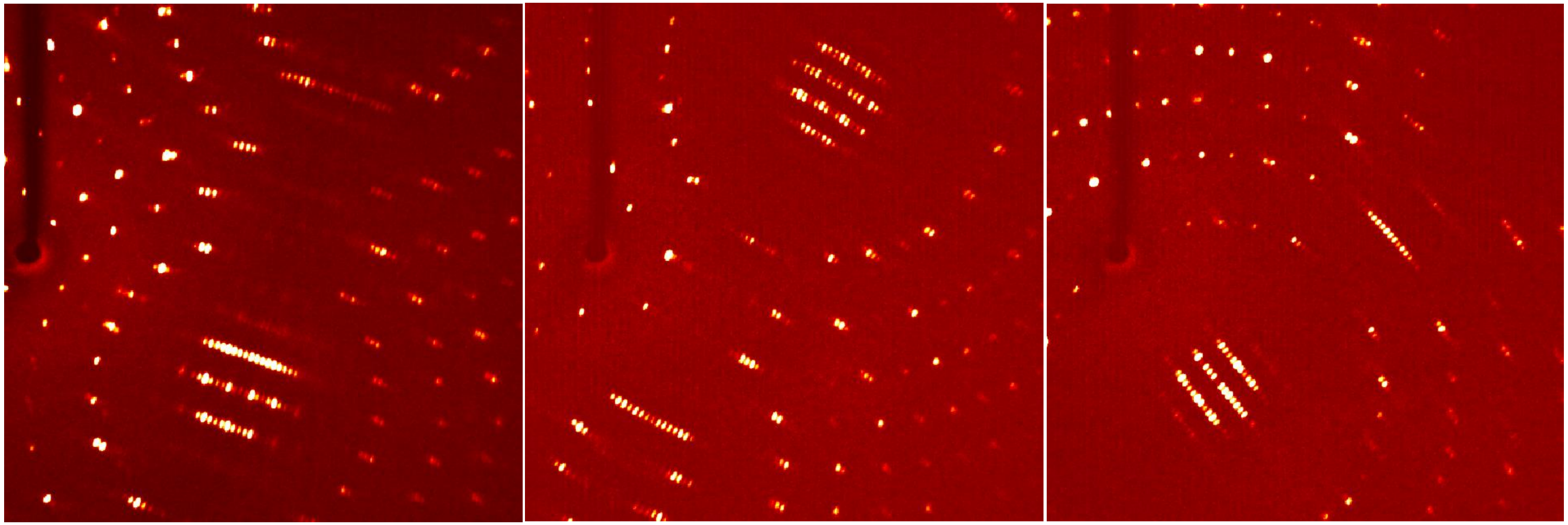

During the X-ray experiment, the detector was moved away (perhaps not far enough) to better separate the nearby reflections due to the large parameter c, but this prevented their "smearing" only along a*. Below are presented the typical frames. It was not possible to find a suitable structural model. Only a layered structure was revealed: four layers per cell, located perpendicular to the c axis. Perhaps the problem was in the incorrect integration of the array of reflections. Integration in automatic mode is carried out incorrectly: a large percentage of rejection of integrated peaks;and, consequently, low completeness of data collection. Apparently, the "box" is too large for integrating reflections.

When choosing a reduced “box” of 0.7x0.7x0.8°, the reflections fall into it almost entirely with good statistical integration parameters and completeness of data collection. Below are the reconstructions of the intensity distribution in the reciprocal space in the hk0, h0l, and 0kl layers. All spots are well indexed. Empirical accounting of absorption for the obtained primary data set is performed well for the diffraction class 4/mmm: (suitable % of reflections rejection, low R_{int}). We also failed to find a structural model with the obtained hklF data (see below).

If you know what needs to be done to correctly conduct the experiment and process the data in order to find the exact solution to the structure, we will be happy to cooperate.

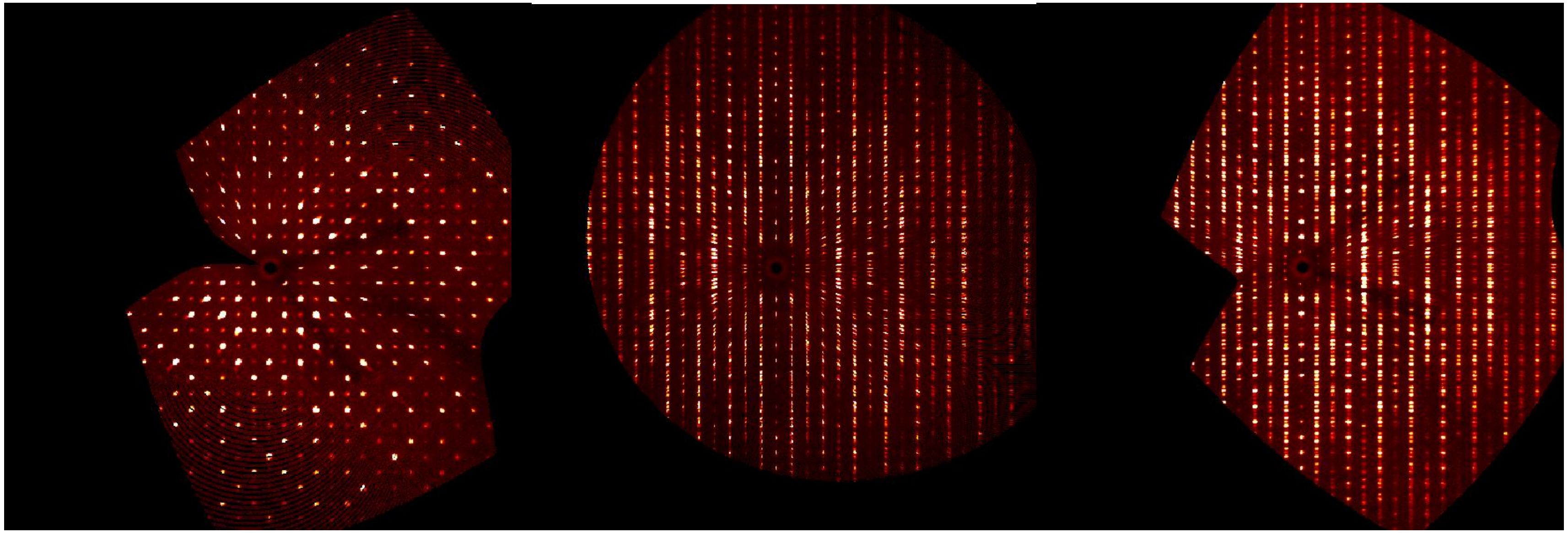

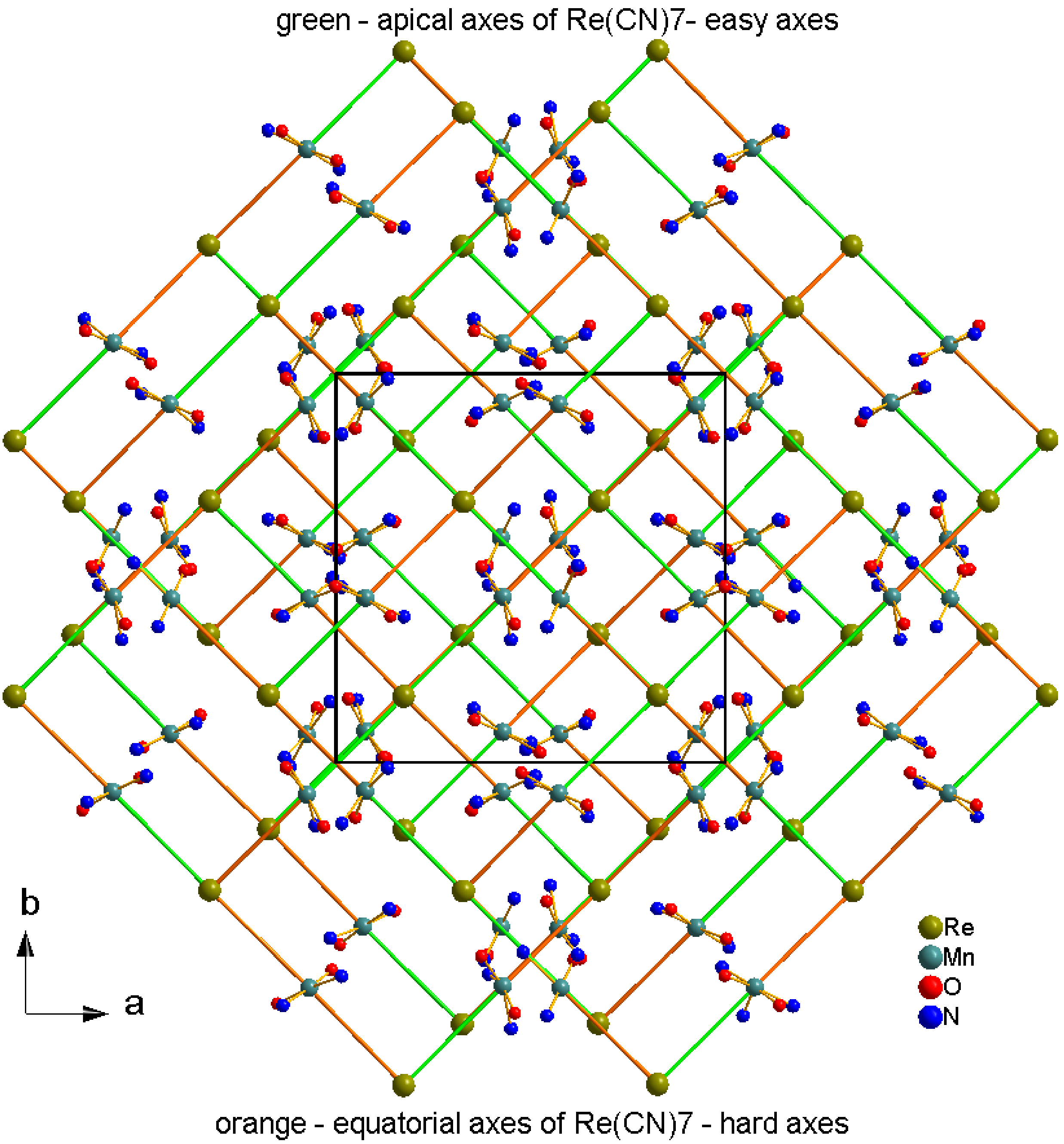

Figure S8. A view of four layers in **2** along *c* - axis. The connections Re-CN_{apical}—{MnN_2O_2} and Re-$CN_{equatorial}$—{MnN_2O_2} are colored in green and orange respectively.

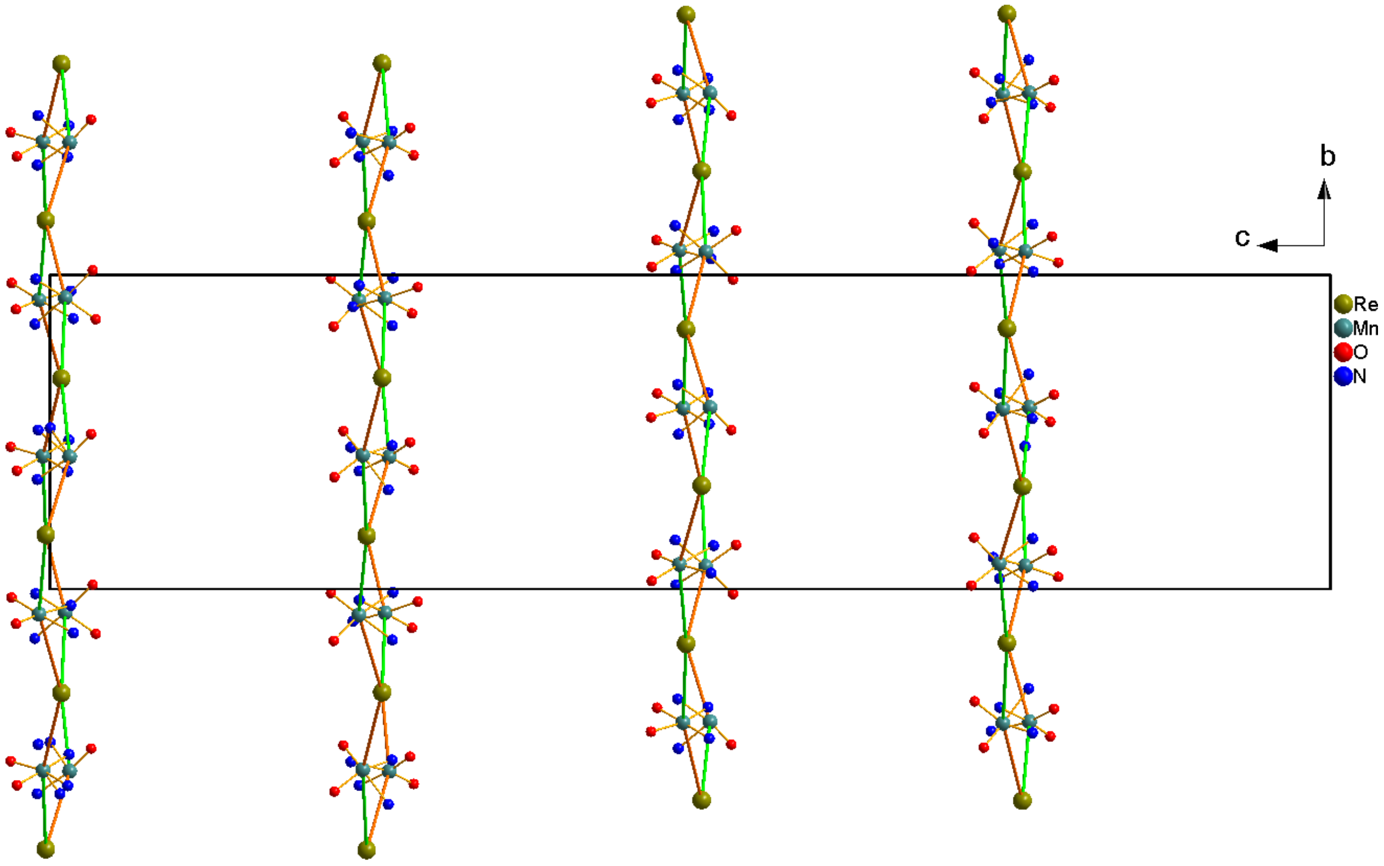

Figure S9. A view of four layers in **2** along *a* - axis. The connections Re-CN$_{apical}$—{MnN_2O_2} and Re-CN$_{equatorial}$—{MnN_2O_2} are colored in green and orange respectively.

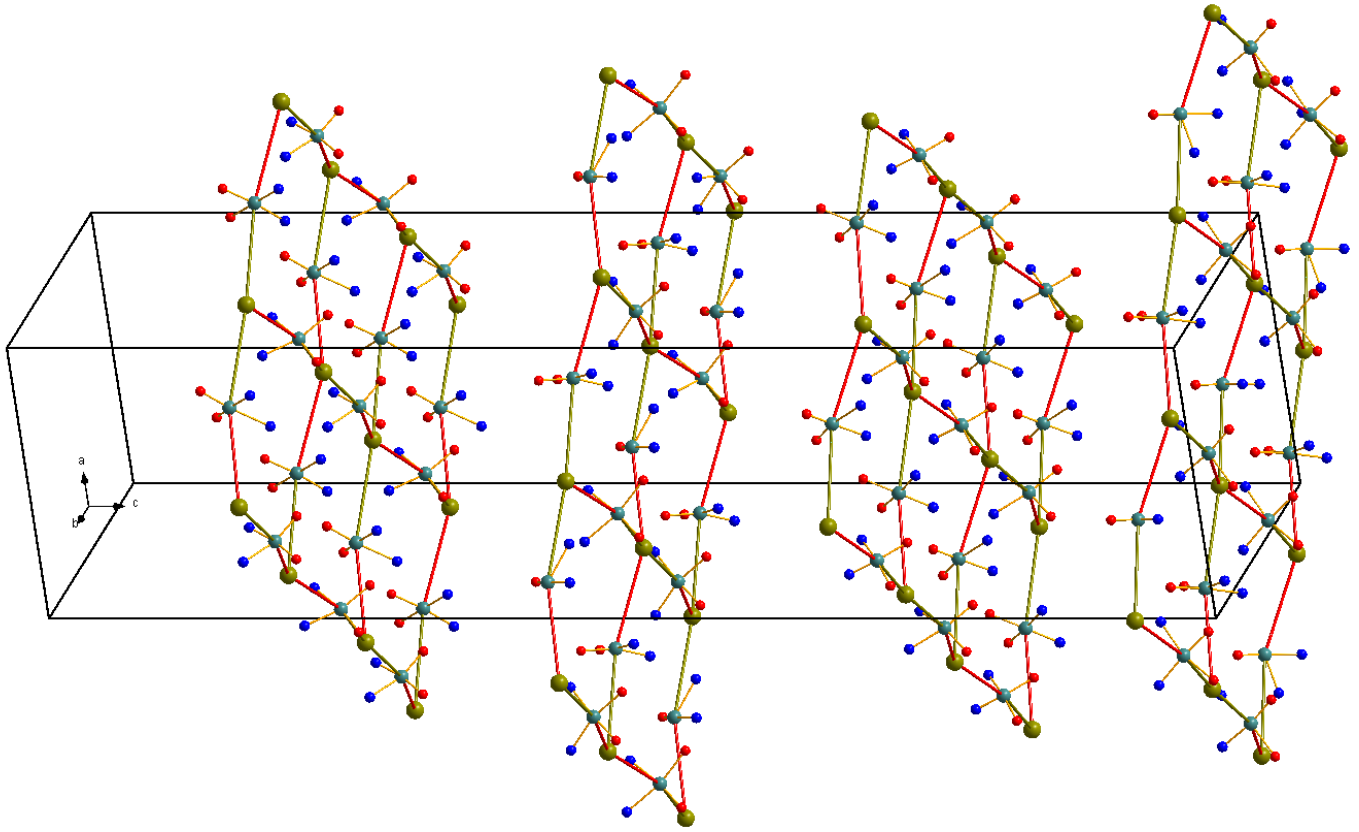

Figure S10. A view of four layers in **2** towards plane hkl = 123. The connections Re-CN$_{apical}$—{MnN_2O_2} and Re-CN$_{equatorial}$—{MnN_2O_2} are colored in olive and red respectively.

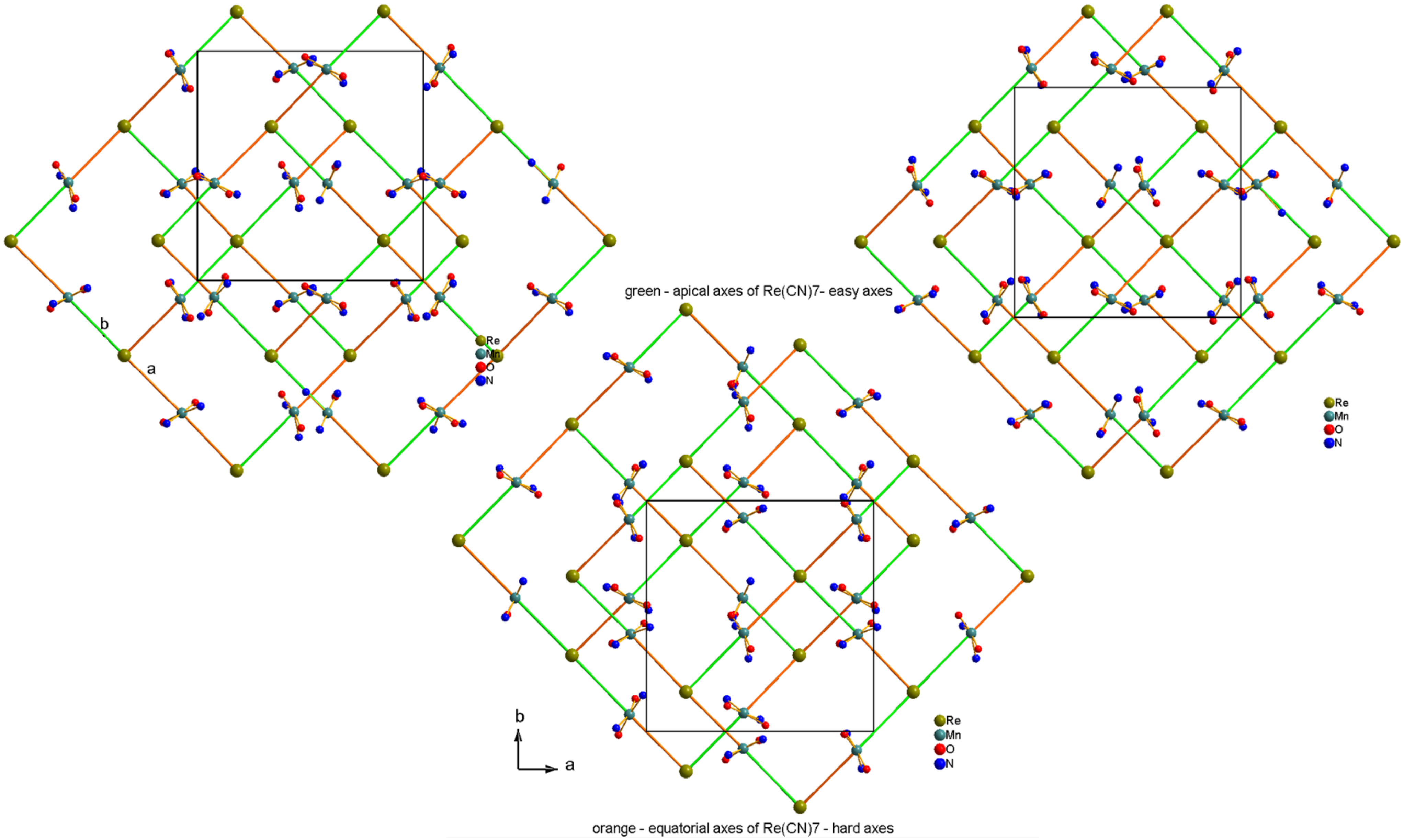

Figure S11. A view of superimposed in pairs layers in for **1**: the first on the second, the second on the third and the third on the fourth**.**

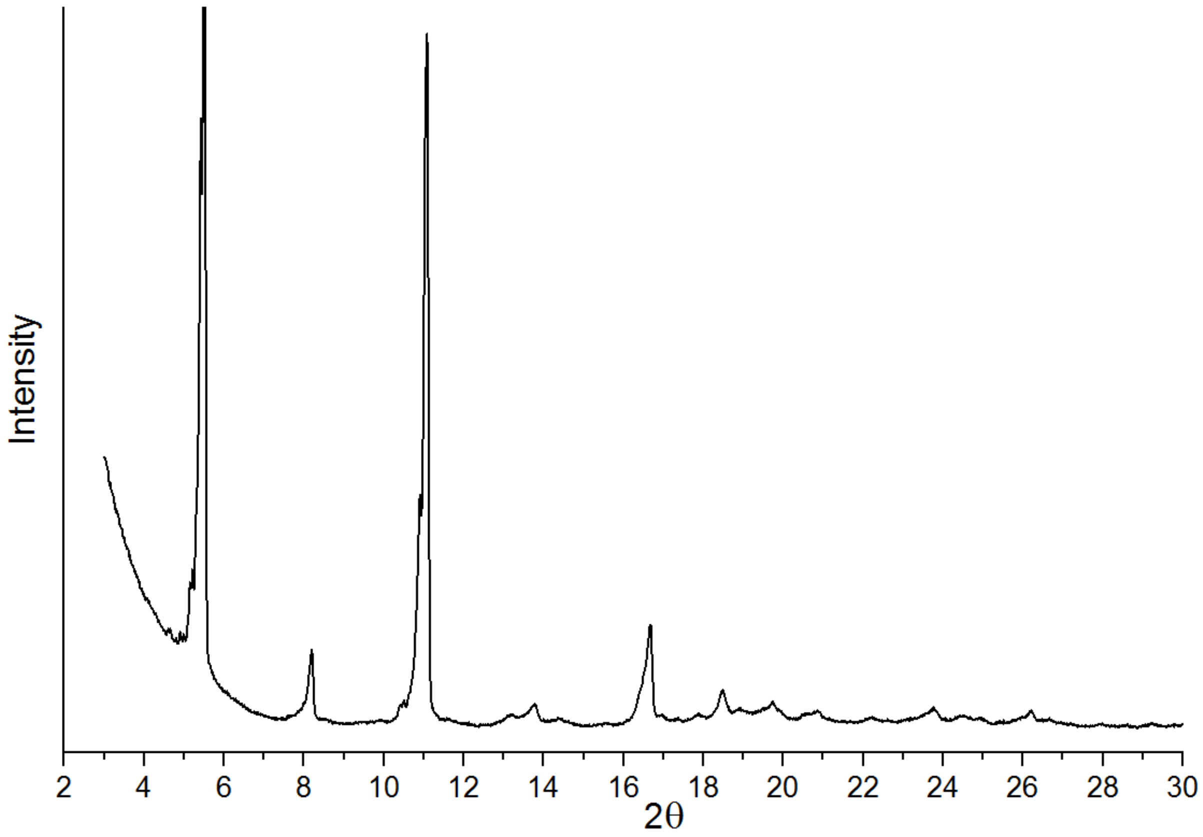

Figure S12. PXRD pattern for the material **1**.

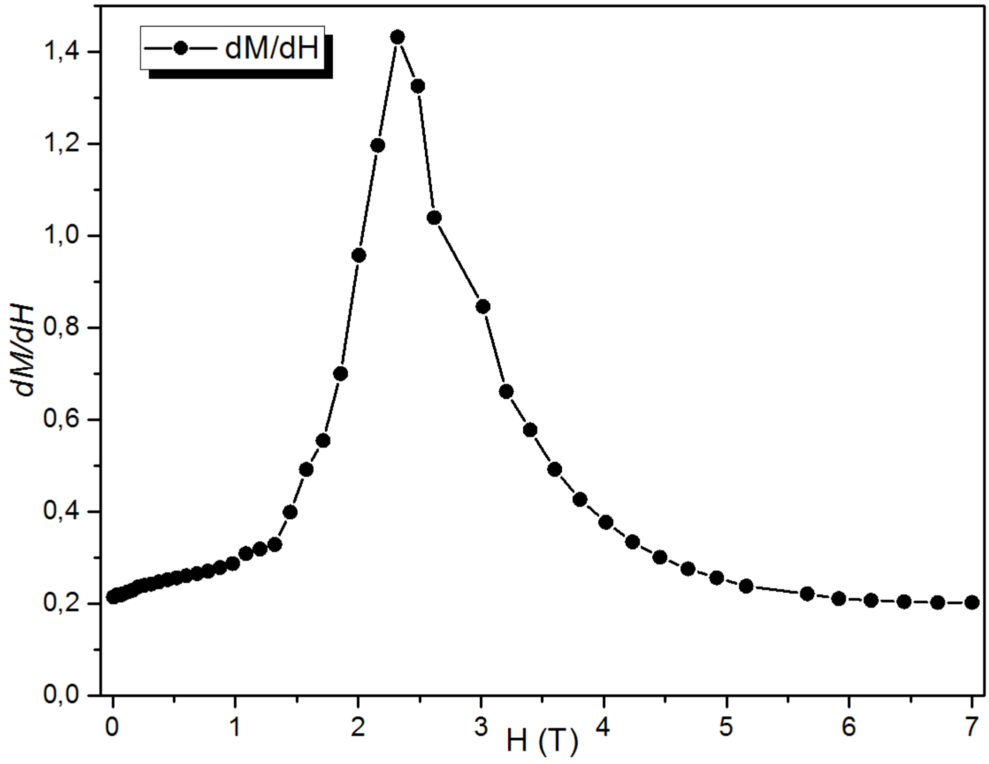

Figure S13. *dM/dH* plot of **2** for the magnetization data collected at 1.8 K.

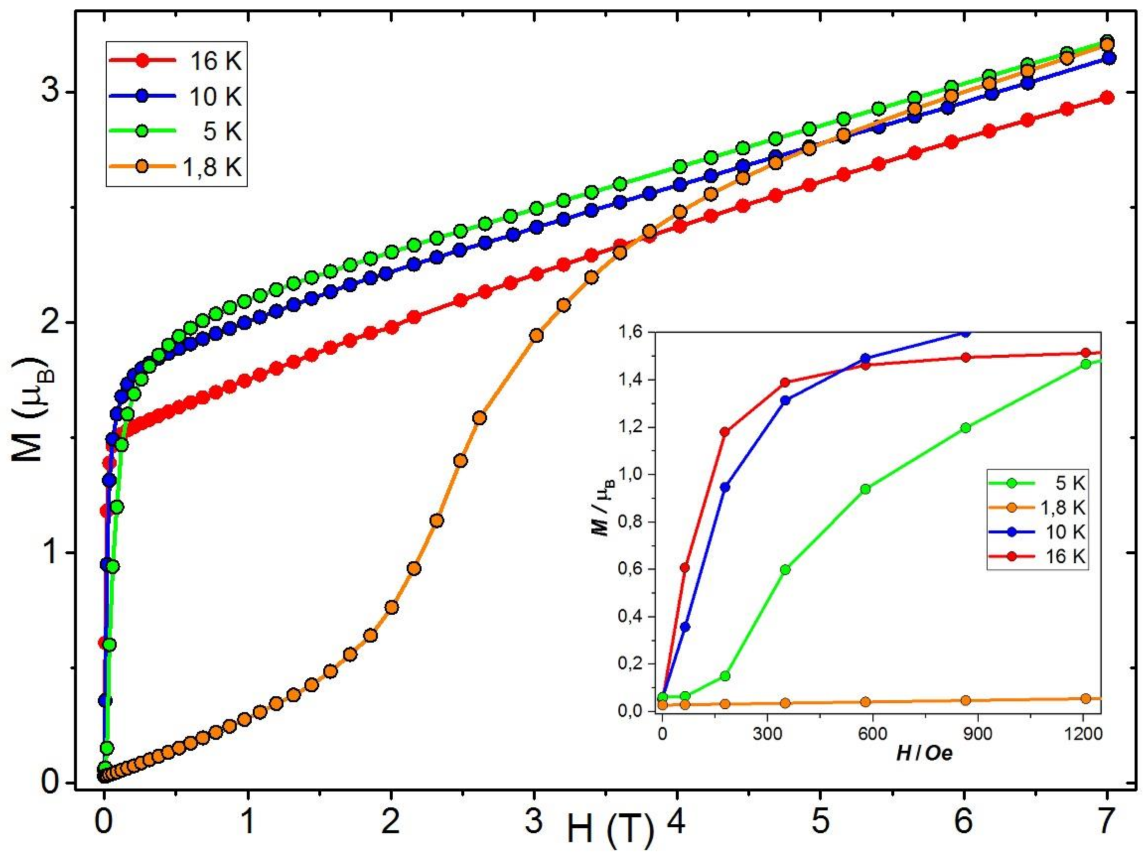

Figure S14. Initial magnetization plots of **2** measured at different temperatures on a polycrystalline sample.

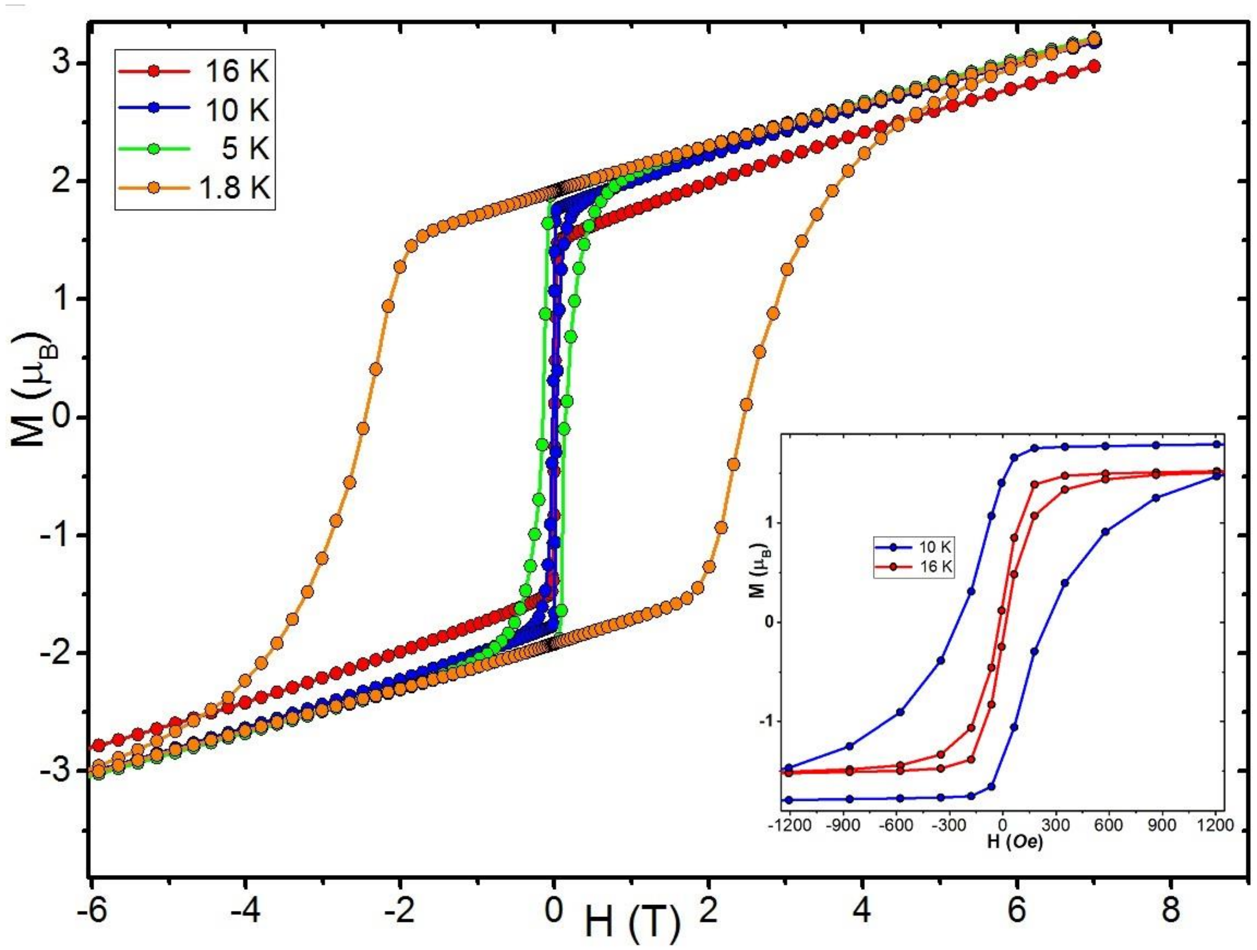

Figure S15. Magnetic hysteresis plots of **2** measured at different temperatures. Inset is a zoom of low field region for the data collected at 10 and 16 K.

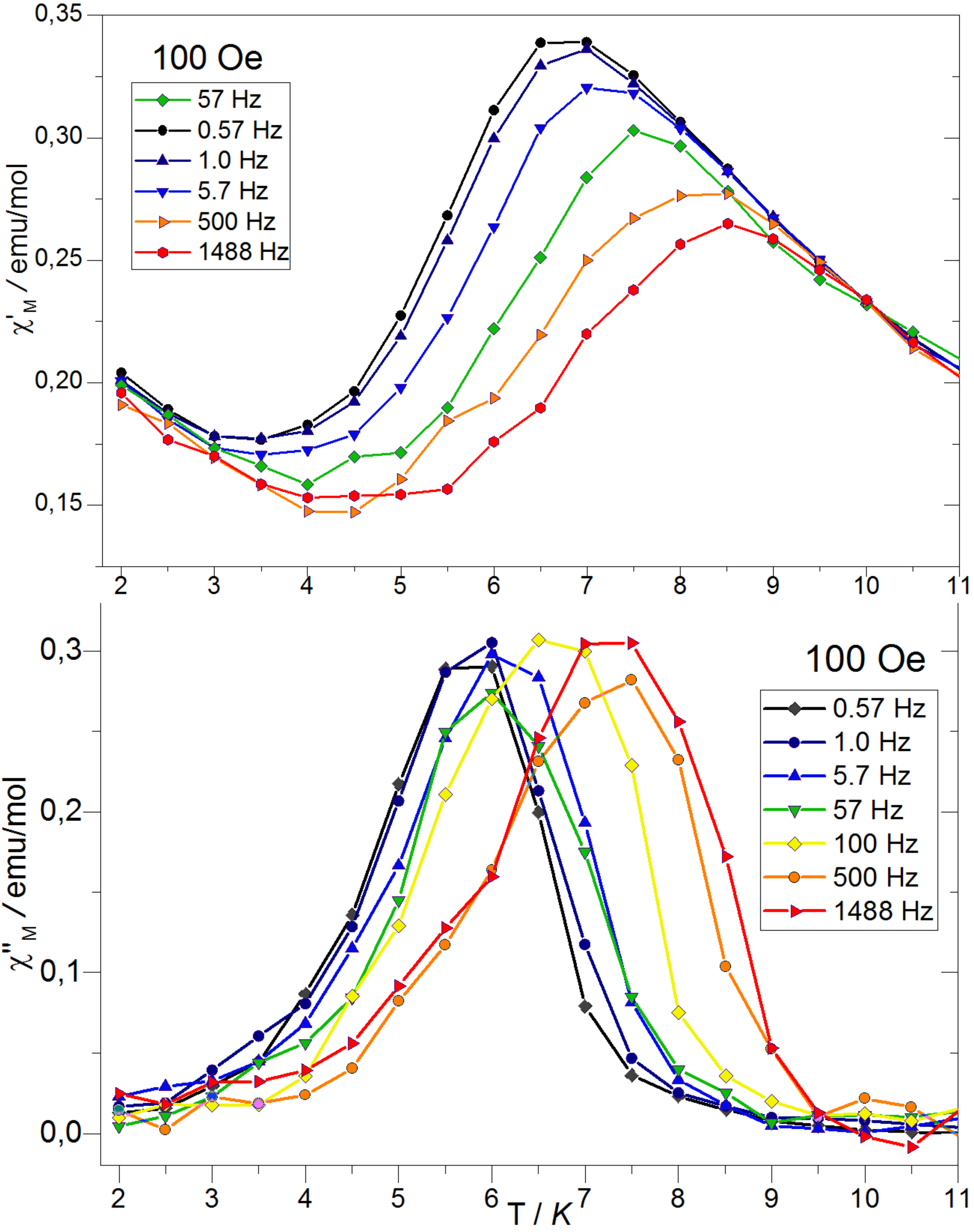

Figure S16. Variable-temperature of the real, χ' (top), and imaginary, χ'' (bottom), parts *ac* molar susceptibility data for **1** under H_{dc} = 100 Oe, H_{ac} = 3 Oe. Solid lines are guides

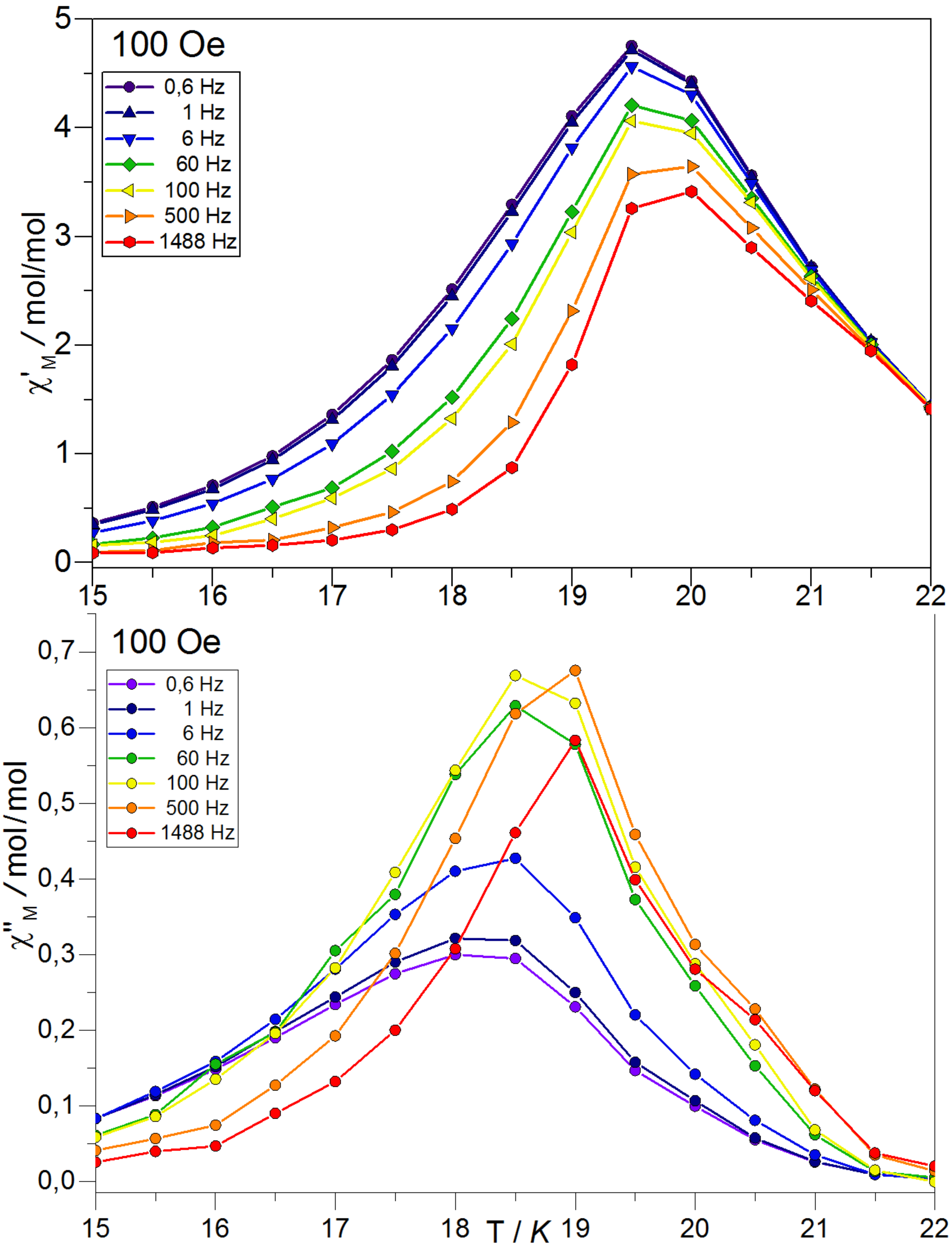

Figure S17. Variable-temperature of the real, χ' (top), and imaginary, χ" (bottom), parts *ac* molar susceptibility data for **2** under H_{dc} = 100 Oe, H_{ac} = 3 Oe. Solid lines are guides.

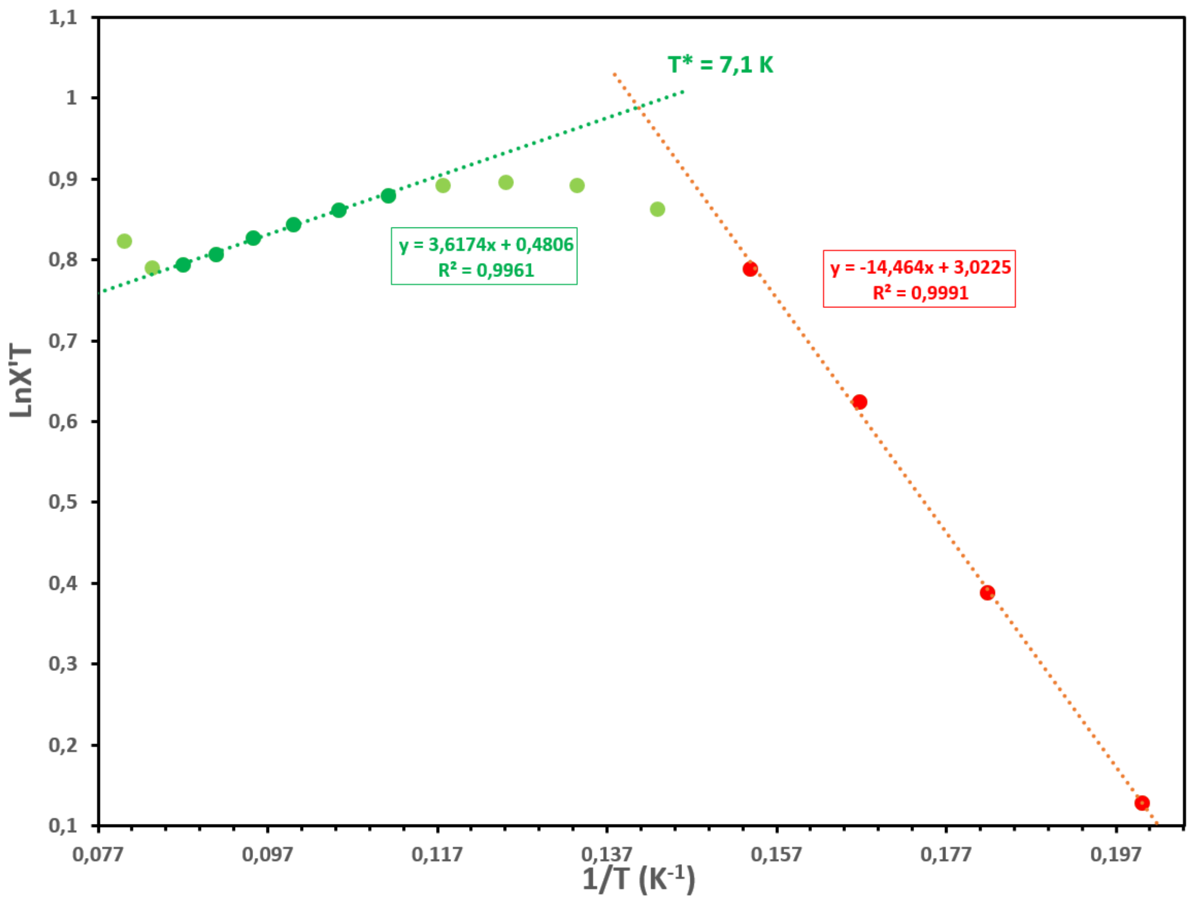

Figure S18. Plots of ln(X′*T*) vs 1/*T* (where X′ is a real component of the *ac* susceptibility for **1** collected in applied *dc* field of 100 Oe, H_{ac} = 3Oe and frequency of 0.56 Hz. The dashed lines correspond to a linear fit.

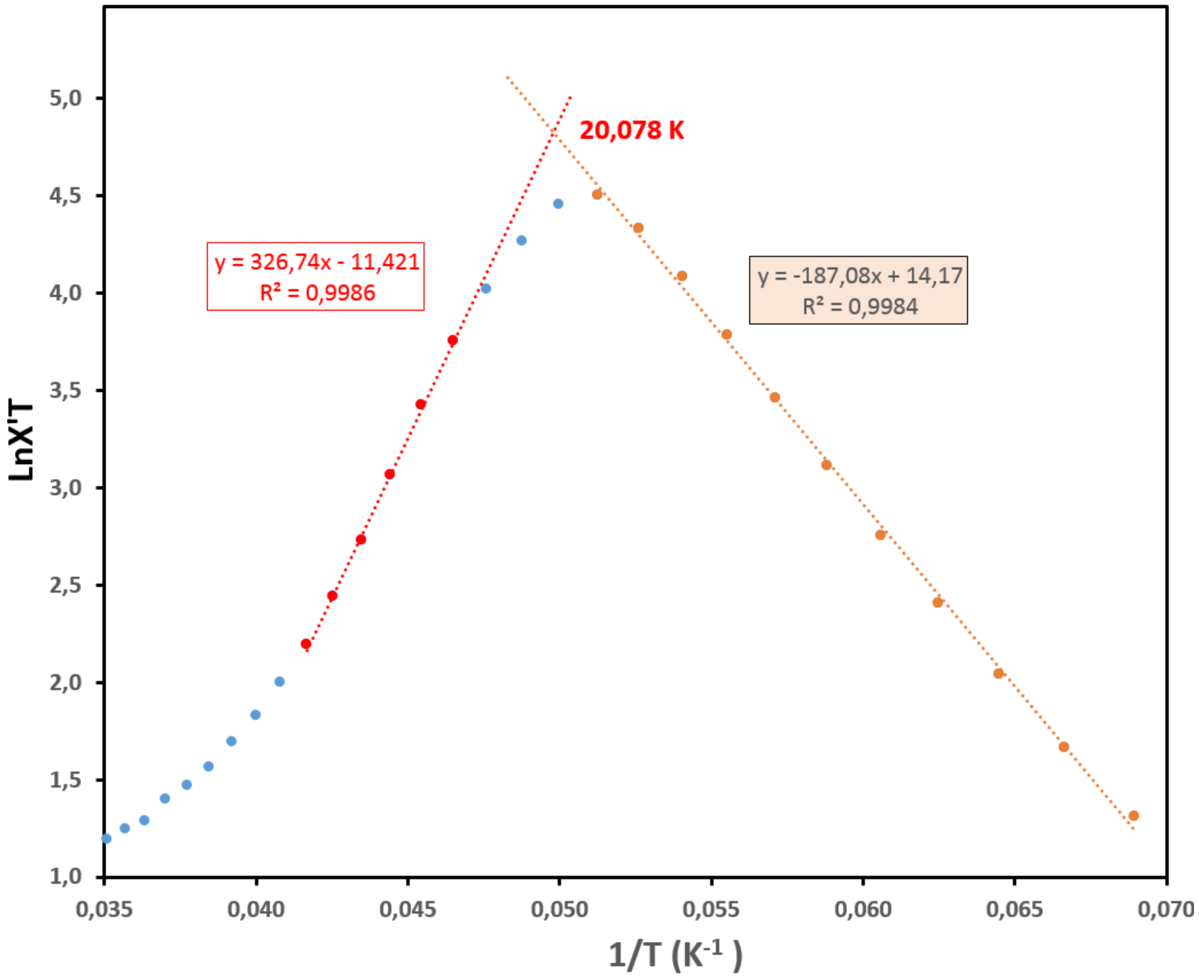

Figure S19. Plots of ln(χ'T) vs 1/*T* (where χ' is a real component of the *ac* susceptibility for **2** collected in applied *dc* field of 100 Oe, H_{ac} = 3Oe and frequency of 0.56 Hz. The dashed lines correspond to a linear fit.
